# CDK-dependent phosphorylation regulates PNKP function in DNA replication

**DOI:** 10.1016/j.jbc.2024.107880

**Published:** 2024-10-11

**Authors:** Fatemeh Mashayekhi, Elham Zeinali, Cassandra Ganje, Mesfin Fanta, Lei Li, Roseline Godbout, Michael Weinfeld, Ismail Hassan Ismail

**Affiliations:** 1 Department of Oncology, Faculty of Medicine & Dentistry, University of Alberta, Edmonton, Alberta, Canada; 2 Biophysics Department, Faculty of Science, Cairo University, Giza, Egypt

**Keywords:** genome instability, FEN1, PNKP, CDKs, DNA replication, Okazaki fragments

## Abstract

Okazaki fragment maturation (OFM) stands as a pivotal DNA metabolic process, crucial for genome integrity and cell viability. Dysregulation of OFM leads to DNA single-strand breaks-accumulation, which is linked to various human diseases such as cancer and neurodegenerative disorders. Recent studies have implicated LIG3-XRCC1 acting in an alternative OFM pathway to the canonical FEN1-LIG1 pathway. Here, we reveal that polynucleotide kinase-phosphatase (PNKP) is another key participant in DNA replication, akin to LIG3-XRCC1. Through functional experiments, we demonstrate PNKP's enrichment at DNA replication forks and its association with PCNA, indicating its involvement in DNA replication processes. Cellular depletion of PNKP mirrors defects observed in OFM-related proteins, highlighting its significance in replication fork dynamics. Additionally, we identify PNKP as a substrate for cyclin-dependent kinase 1 and 2 (CDK1/2), which phosphorylates PNKP at multiple residues. Mutation analysis of these phosphorylation sites underscores the importance of CDK-mediated PNKP phosphorylation in DNA replication. Our findings collectively indicate a novel role for PNKP in facilitating Okazaki fragments joining, thus shedding light on its contribution to genome stability maintenance.

DNA replication is a vital procedure in all living organisms, ensuring the accurate duplication of cellular genetic information before cell division occurs, producing two genetically identical daughter cells. Within eukaryotes, this replication process is confined to the S phase (1, 2, 3). Replicating eukaryotic chromosomal DNA demands stringent control mechanisms to guarantee that each cell's genome undergoes replication precisely once per cell cycle, thereby minimizing errors (4, 5). Cyclin-dependent kinases (CDKs) meticulously regulate the DNA replication process by phosphorylating various replication factors, enabling their entry into the S phase, and facilitating DNA synthesis (6). A failure in this regulatory system can have severe consequences, including the onset of diseases such as cancer (7, 8).

DNA replication involves a semi-discontinuous mechanism (3). The leading-strand DNA synthesis, facilitated by DNA polymerase ε (Pol ε), proceeds continuously, whereas the lagging-strand DNA synthesis occurs discontinuously (9, 10). In the latter process, a complex comprising DNA polymerase α (Pol α) and primase synthesize an RNA primer (7–14 nt) along with a short stretch (10–20 nt) of DNA (9, 10). Subsequently, the Pol α/primase complex is replaced by DNA polymerase δ (Pol δ), which extends the RNA–DNA primers, generating discrete DNA fragments termed Okazaki fragments. The maturation of these fragments involves a series of coordinated reactions catalyzed by polymerases, nucleases, and DNA ligases. Initially, the nascent DNA synthesized by Pol δ from the upstream Okazaki fragment extends into the downstream fragment, creating a 5′ flap structure encompassing the RNA–DNA primer (11, 12). This structure is cleaved by members of the RAD2 structure-specific endonuclease family, such as flap endonuclease 1 (FEN1), to generate a ligatable DNA nick, which is then sealed by DNA ligase I (LIG I) (13, 14, 15). Despite the efficiency of the canonical FEN1/LIG I pathway in Okazaki fragment maturation (OFM), biochemical experiments suggest that approximately 15 to 30% of human POLδ molecules disengage before reaching the downstream Okazaki fragment, even in the presence of the proliferating cell nuclear antigen (PCNA) processivity factor (16). Considering that each human S phase involves the formation of 30 to 50 million Okazaki fragments, failure to ligate even a small fraction of these intermediates could lead to a significant number of single-strand breaks (SSBs) or gaps in nascent DNA strands (17). Interestingly, unligated Okazaki fragments that evade canonical processing are recognized by poly(ADP-ribose) polymerase 1 (PARP1), initiating a backup pathway involving SSB repair proteins XRCC1 and LIG3 (17, 18). This PARP1-dependent signalling and repair mechanism likely contributes to maintaining the integrity of nascent DNA strands during DNA replication. However, it remains uncertain whether XRCC1 and LIG3 are the sole SSB repair proteins involved in OFM.

Polynucleotide kinase 3′-phosphatase (PNKP) is a key enzyme that operates in the base excision repair (BER)/SSB repair (SSBR) and non-homologous end joining (NHEJ) repair pathways. It serves as a dual-function end-processing enzyme, carrying out phosphorylation of DNA 5′-hydroxyl termini and dephosphorylation of DNA 3′-phosphate termini (19, 20, 21). The human PNKP enzyme spans 521 amino acids (22). It comprises an amino-terminal region containing the forkhead-associated (FHA) domain (amino acid residues 1–110). Additionally, it includes phosphatase (amino acid residues 146–337) and kinase (amino acid residues 341–516) domains in the carboxy-terminal region. The enzymatic domains are linked by a linker region (amino acid residues 111–145) to the FHA domain (22, 23). PNKP mutations are associated with several human inherited disorders, including microcephaly and seizures (MCSZ), characterized by neurodevelopmental abnormalities, as well as ataxia oculomotor apraxia 4 (AOA4), and Charcot-Marie-Tooth disease (CMT2B2), both of which manifest as neurodegenerative conditions (24, 25, 26). The generation of a conditional PNKP knockout mouse revealed that PNKP plays a critical role in the genome maintenance of adult progenitor cells (27). The primary substrate for PNKP is single-stranded DNA gaps, commonly encountered as intermediates in OFM.

In this study, we unraveled a novel role for PNKP during DNA replication. In agreement with others, we found that PNKP interacts with PCNA, accumulates on nascent DNA during DNA replication, and phosphorylates OF-like DNA termini. The loss of PNKP culminates in cellular phenotypes reminiscent of OFM problems. Moreover, our data suggest this new role of PNKP is regulated by CDK1/2 phosphorylation on linker and phosphatase domains.

## Results

### PNKP localizes to the replication fork (RF) and interacts with PCNA

**Figure 1:**
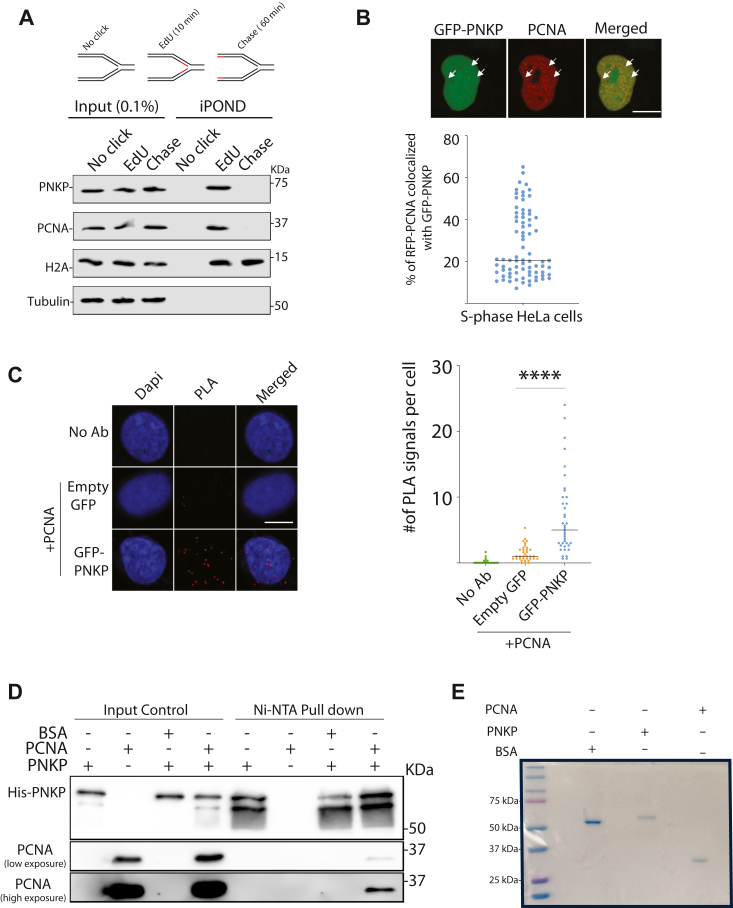
**PNKP is at the RF and associates with newly synthesized DNA and PCNA.***A*, (*Top*) schematic of the iPOND labelling protocol. (*Bottom*) HEK293T cells were labelled with EdU for 10 min prior to performing the iPOND protocol. The EdU-containing medium was removed, and cells were washed once before incubating for 60 min in a medium containing 10 μM thymidine (chase) before performing iPOND. iPOND eluates were immunoblotted using PNKP, PCNA, tubulin and histone H2A antibodies. No-click control indicates that the sample was processed with no biotin-azide. *B*, localization of GFP-PNKP with PCNA. Representative images are shown (*top*), and quantifications of the percentage of GFP-PNKP foci colocalizing with PCNA are shown (*bottom*). More than 70 cells in each condition were analyzed. The graph represents the data from two independent replicates. *Arrowheads mark* PNKP foci colocalizing with PCNA in S phase cells. *C*, PLA assay with U2OS cells shows the interaction between PCNA and PNKP. U2OS cells were transfected with either GFP or GFP-PNKP plasmids 24 h before the experiment. Antibodies against GFP and PCNA were used. No antibody was used for the negative control (no Ab). Three independent experiments were performed, and more than 30 cells were analyzed in each experiment. The graph represents the data from three independent replicates. For quantifications, two-tailed, unpaired, non-parametric student’s t-tests were performed in Prism to determine statistical significance. The median values are marked on the graph. *Asterisks* depict statistically significant differences: ∗∗∗∗ (*p* < 0.0001). *D*, pull down experiment with purified His-PNKP and PCNA. Bovine serum albumin (BSA) was used as a negative control. n = 2 independent experiments were performed, and representative blots are shown. *E*, 1 μg purified proteins used in (*D*) were run on a 10% polyacrylamide gel and stained with Coomassie blue to confirm their purity. The scale bar is 10 μm.

Previous nascent chromatin capture proteomics studies indicated, but did not confirm, the enrichment of PNKP at the RF, supporting the notion that PNKP might play an essential role in unperturbed DNA replication (28, 29). To test this hypothesis, we first examined the enrichment of PNKP at the DNA RF by carrying out the Isolation of Proteins On Nascent DNA (iPOND) assay under unperturbed conditions. iPOND is a robust method for detecting proteins at sites of newly replicated DNA (30). The iPOND assay employs “click” chemistry to attach biotin to a nucleoside analog (EdU) that is integrated into newly synthesized DNA (30). This enables the examination of proteins linked with RFs. As observed previously, PCNA was detected at elongating RFs, and its levels decreased in the thymidine chase sample (Fig. 1*A*). The equal level of histone H2A detected on isolated chromatin (Fig. 1*A*) indicates that an equivalent amount of EdU-labeled DNA was purified in each sample. We detected endogenous PNKP in iPOND eluates but not in controls lacking the “click” reagent (Fig. 1*A*), suggesting that PNKP associates with the nascent DNA at the progressing fork. Thymidine chase resulted in the loss of PNKP in iPOND samples (Fig. 1*A*), providing additional evidence that PNKP is *a bona fide* RF-associated protein. We next used confocal microscopy to study the colocalization of PNKP with PCNA in replicating cells. GFP-PNKP and RFP-PCNA fusion proteins were co-expressed in HeLa cells and assayed for their colocalization using confocal microscopy. PCNA foci-positive cells, representing S-phase, were chosen to quantify PNKP and PCNA foci colocalization. In unperturbed cells, PNKP forms nuclear foci that colocalize with PCNA (Fig. 1*B*), consistent with a previous study (31). Moreover, by using a proximity ligation assay (PLA) (32) with GFP and PCNA-specific antibodies, we visualized the interaction of exogenously expressed GFP-PNKP and PCNA (Fig. 1*C*). The PLA signal in cells expressing GFP-PNKP was significantly higher than the ones expressing GFP indicating a specific interaction between PNKP and PCNA (Fig. 1*C*). Together, the data strongly support the existence of PNKP at the RF and the interaction of PNKP with PCNA at active RFs. These data raise the question of whether PNKP directly interacts with PCNA. To investigate this, we carried out an *in vitro* binding experiment with purified His-tagged PNKP and PCNA (Fig. 1*D*). Intriguingly, the results revealed that PNKP directly interacts with PCNA, as we found PCNA in the pulled-down PNKP sample (Fig. 1*D*). No observed interaction between PNKP and bovine serum albumin (BSA) as control suggested that PNKP interacts with PCNA specifically. To ascertain the purity of the purified proteins used in Figure 1*D*, we subjected 1 μg of each protein to electrophoresis on a 10% polyacrylamide gel, as illustrated in Figure 1*E*. The obtained results affirm the integrity and purity of the proteins under examination.

### PNKP-deficient cells display longer nascent DNA tract length

**Figure 2:**
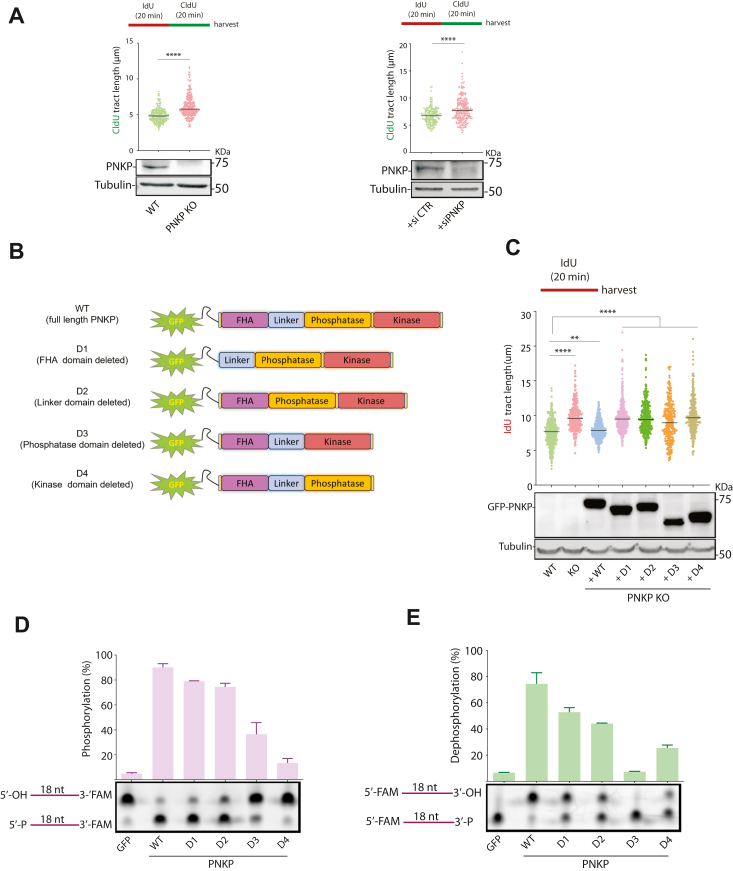
**Increased nascent DNA tract length in PNKP KO cells.***A*, DNA fiber experiments showing faster replication fork progression in HCT116 PNKP KO cells compared to wild-type (WT) (*left*) and in U2OS cells transfected with siRNA against PNKP (siPNKP) compared to control siRNA (siCTR) (*right*) under unperturbed conditions. The quantification of the CldU tract length is presented, with the median values marked on the graph. Between 100 to 300 fibers were measured per condition. The graph is representative of pulled data from at least three independent experiments. *Asterisks* indicate statistical significance (Mann–Whitney test, two-sided): ∗∗∗∗ (*p* < 0.0001). A schematic representation of the assay conditions is presented at the top of each graph. Immunoblot showing the expression levels of PNKP is shown below. Tubulin serves as a loading control. *B*, Schematic depiction of the GFP-tagged PNKP deletion mutants used in *C*. *C*, DNA fibre experiments using PNKP KO2 cells reconstituted with PNKP WT or PNKP deletion mutants shown in (*B*). The quantification of the ldU tract length is presented, with the median values marked on the graph. Between 140 to 400 fibres were measured per condition. The graph is representative of pulled data from three independent experiments. *Asterisks* indicate statistical significance (Mann–Whitney test, two-sided): ∗∗ (*p* < 0.01), ∗∗∗∗ (*p* < 0.0001). A schematic representation of the assay conditions is presented at the top of the graph. Immunoblot showing the expression levels of each PNKP expression construct is shown below. Tubulin serves as a loading control. *D*, PNKP kinase assay. A 3′- FAM-labelled 18-nt DNA substrate bearing a 5′-OH terminus was incubated with either immunoprecipitated PNKP WT or PNKP mutants depicted in (*B*), in the presence of 0.2 mM ATP followed by gel electrophoresis analysis. Cells transfected with GFP construct (GFP) were used as a negative control. *E*, PNKP phosphatase assay. A 5′-FAM-labeled 18-nt DNA substrate with a 3′ phosphate group was incubated with either PNKP WT or PNKP mutants depicted in (*B*) before analyzing by gel electrophoresis. Cells transfected with the GFP construct (GFP) were used as a negative control.

**Figure 3:**
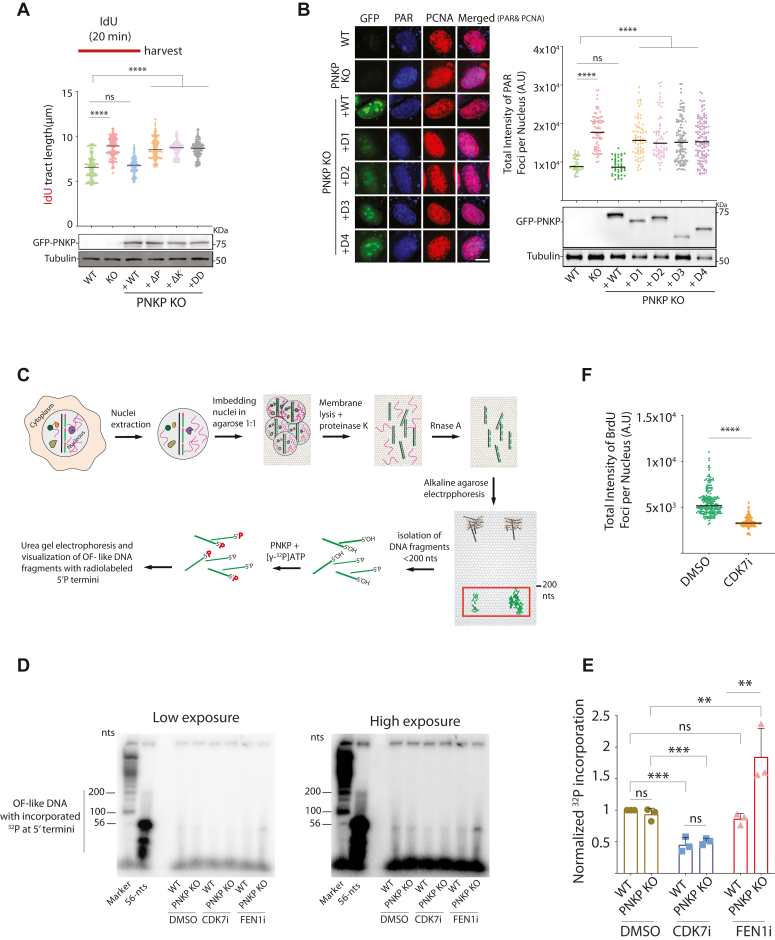
**Loss of PNKP results in the accumulation of OF-like DNA structures with 5′-OH termini.***A*, DNA fiber experiments using PNKP KO2 cells reconstituted with PNKP WT or PNKP point mutants lacking either phosphatase (ΔP^−^), kinase (ΔK^−^), or phosphatase and kinase (DD) activities. The quantification of the ldU tract length is presented, with the median values marked on the graph. At least 180 fibers were measured per condition. The graph is representative of pulled data from two independent experiments. *Asterisks* indicate statistical significance (Mann–Whitney test, two-sided): ns (non-significant), ∗∗∗∗ (*p* < 0.0001). A schematic representation of the assay conditions is presented at the top of the graph. Immunoblot showing the expression levels of each PNKP expression construct is shown below. Tubulin serves as a loading control. *B*, immunofluorescence experiment showing increased PAR chain formation (indicative of defective OFM) in PNKP KO1 cells reconstituted with different truncation mutants as in *B*. Cells were treated with a PARG inhibitor (PARGi) 10 μM, for 20 min before fixation to block PAR chain removal. Representative images of cells are shown on the *left*. Quantification of the PAR signal in PCNA positive cells is shown on the *right*. At least 200 cells were analysed for each condition. Similar results were obtained from two independent experiments. The median values are marked on the graph. The immunoblot experiment showing the expression levels of each PNKP expression construct is shown below. Tubulin serves as a loading control. To determine statistical significance, unpaired Student’s t-tests were performed in Prism. *Asterisks* depict statistically significant differences: ns (non-significant), ∗∗∗∗ (*p* < 0.0001). *C*, schematic representing the protocol applied in *D* to isolate OF-like DNA fragments and radio-label them with purified human PNKP and ^32^P-ATP. Briefly, nuclei were extracted and embedded in agarose gel prior to the lysis of the nuclear membrane to avoid DNA sheering. After removing RNA, the gel plugs were run on an alkaline agarose gel. DNA fragments smaller than 200mer were isolated from the gel, labeled with PNKP and ^32^P-ATP, and run on a denaturing urea gel. *D*, unchallenged HCT116 cells were treated with either CDK7 inhibitor (CDK7i) or FEN1 inhibitor (FEN1i). DMSO treatment was used as a control. Nuclear DNA was run on an alkaline gel and ≤200 nt DNA fragments were isolated and labeled with ^32^P-ATP by exogenous PNKP. The panels representing radiolabeled DNA fragments after electrophoresis on an 8% urea gel. *E*, the graph represents three independent replicates. The signal intensity of the whole smear was measured for each sample. The background signal was deducted from the signals of the samples, and the resultant values were subsequently normalized to those of the wild-type DMSO. Two-way ANOVA with Tukey’s multiple comparisons was performed in Prism. *Asterisks* depict statistically significant differences: ns (not significant), ∗∗ (*p*<<0.01), ∗∗∗ (*p* < 0.001). *F*, cells were treated with CDK7 inhibitor (CDK7i), or DMSO, for 80 min. At the last 10 min of CDK7i incubation, BrdU was added to label replicating DNA. The graph represents three independent experiments. Asterisks indicate statistical significance (Mann–Whitney test, two-sided): ∗∗∗∗ (*p* < 0.0001). The scale bar is 10 μm.

To investigate PNKP's involvement in DNA replication, we evaluated the effects of completely deleting PNKP. Initially, we utilized CRISPR/Cas9 gene editing to disrupt the expression of native PNKP in HeLa (designated PNKP KO1) and HCT116 (PNKP KO2) cell lines (33) We explored the function of PNKP in RF progression using the DNA fiber assay, a technique that allows for the detailed investigation of DNA replication dynamics at the level of individual molecules (34). In this assay, cells were initially treated with two different halogenated thymidine analogs, namely IdU (5-iodo-2′-deoxyuridine) and CldU (5-chloro-2′-deoxyuridine), sequentially marking the initiation/early elongation phases and subsequent progression of RFs at active origins (34). After spreading DNA fibers onto microscopic slides, newly synthesized DNA regions were identified using fluorescent dye-labeled antibodies that specifically bind to the incorporated thymidine analogs. The length of fluorescence signals observed in captured micrographs was then utilized to calculate the length of the replicated DNA tracts. Under unperturbed growth conditions, cells lacking PNKP (PNKP KO2) as well as deficient in PNKP (U2OS cells + siPNKP) showed longer nascent tract lengths (Fig. 2*A*). Interestingly, reconstituting PNKP KO2 cells with PNKP WT rescued nascent tract length to normal levels. In contrast, rescuing PNKP KO2 cells with PNKP constructs lacking the FHA domain (D1, residue 2–110 deleted), linker domain (D2, residue 111–145 deleted), phosphatase domain (D3, residue 146–337 deleted), or kinase domain (D4, residue 338–521 deleted) failed to restore the nascent tract length to normal levels (Fig. 2, *B* and *C*). Our flow cytometry data demonstrated that the differences in nascent DNA tract length are not due to changes in the cell cycle, as the percentages of cells in the S and G2 phases of the cell cycle were similar under all conditions (Fig. S1*A*). A considerable impact on the enzymatic activities of PNKP is expected in PNKP truncated mutants. Therefore, the kinase and phosphatase activities of the various PNKP mutants (D1-D4) were analyzed to evaluate the effects of the truncation on PNKP enzymatic activities. As indicated in Figure 2, *D* and *E*, D1 (lacking the FHA domain) and D2 (lacking the linker domain) mutants maintained both phosphatase and kinase activities. However, as expected, removing either the phosphatase or kinase domain dramatically reduces the enzymatic activities of PNKP (Fig. 2, *D* and *E*). To further confirm the role of phosphatase and kinase activities of PNKP in DNA replication fork speed, we tested whether PNKP point mutants lacking phosphatase activity (D171A and D173A, ΔP^−^) or kinase activity (K378A, ΔK^−^) or both (DD) would be able to restore tract length in PNKP KO cells (35). Consistent with our truncation mutant results, we found that reconstituting PNKP KO2 cells with any of the PNKP point mutants failed to restore tract length to normal levels indicating that PNKP phosphatase and kinase activities are required for its function in DNA replication (Fig. 3*A*). These data indicate that multiple activities/domains of PNKP are necessary for DNA RF dynamics.

### PNKP ensures 5′-P DNA ends on Okazaki fragment (OF)-like DNA

The ∼20% increase in tract length observed in PNKP-deficient cells is reminiscent of cells lacking DNA replication factors involved in OFM such as FEN1 and LIG1 (36, 37). Consistent with previous reports, we also observed that cellular depletion of FEN1 or LIG1 results in longer nascent DNA tracts (36, 37) (Fig. S1*B*). The similarities in RF defects observed in cells lacking factors involved in OFM and PNKP KO cells led us to investigate whether PNKP-deficient cells have defects in OFM. Recent studies have indicated that the inactivation of LIG1 or FEN1 increased S phase poly(ADP-ribose) (PAR) polymers upon inhibition of PAR glycohydrolase (PARG) by a specific inhibitor (PARGi) (18). As increased chromatin PAR chain formation represents a marker of OFM defects (18), we measured PAR chain formation in PNKP-deficient cells treated with an inhibitor to PARG. Similar to LIG1 depletion, PAR chain formation was enhanced in PNKP-deficient cells (Fig. 3*B*). To understand which domain(s) of PNKP is required for OFM, PAR chain formation was examined in cells lacking PNKP compared to PNKP KO1 cells expressing one of the truncated mutants (D1-D4) (Fig. 3*B*). The results indicated that all domains of PNKP serve essential roles in OFM, as none of them could rescue the observed PAR chain phenotype seen in PNKP KO2 cells (Fig. 3*B*). As a control, we found that the PARP inhibitor Olaparib impaired the formation of PAR signal (Fig. S1*C*). To confirm that the accumulation of unligated Okazaki fragments causes PAR chain formation, their immunofluorescence detection was inhibited by treatment with Emetine, an inhibitor of Okazaki fragment synthesis (38) (Fig. S1*D*).

The requirement of PNKP for OFM raised the intriguing possibility that OFs with 5′-OH termini accumulate in cells, an end requirement that explains the necessity for PNKP enzymatic activity. Consistent with this hypothesis, previous reports (39, 40) isolated OF-like DNA with 5′-OH termini from unperturbed cells, implying a novel step in DNA synthesis. Therefore, we sought to confirm the accumulation of OF-like fragments with DNA 5′-OH termini in unperturbed cells and examine the contribution of PNKP in this process. In pursuit of this objective, as illustrated in Figure 3*C*, we extracted nuclei from S-phase synchronized PNKP WT and PNKP KO2 cells and embedded them in agarose plugs as a preventive measure against DNA shearing prior to lysis treatment. Exposed DNA in agarose plugs devoid of RNA was then denatured and run on a 1.5% alkaline agarose gel. Next, DNA fragments of ≤200 nucleotides (expected size of OFs) were isolated from the agarose gel, and the accumulated 5′-OH DNA ends were visualized by incubating the isolated DNA with radiolabelled ATP ([γ-^32^P] ATP) and purified human PNKP before analysis on an 8% denaturing urea gel. While T4 PNKP is known to phosphorylate both RNA and DNA 5′-OH ends and to hydrolyze RNA or DNA 3′-phosphate ends, in contrast, mammalian PNKP has only been shown to phosphorylate DNA *in vitro* (41, 42, 43, 44, 45). The ^32^P radioactive signal represents the abundance of OF-like DNA fragments with 5′-OH termini that PNKP has converted to 5′-phosphate. To validate the DNA replication dependence and connection with the OFM process of these labeled fragments, we initially verified the reliance on DNA replication for the generation of 5′-OH DNA fragments. This was achieved by using a CDK7 inhibitor (CDK7i) to hinder new-origin firing (46). Mammalian cells can use the LIG3/XRCC1 complex to join Okazaki fragments when FEN1/LIGI is absent (18). Considering the potential involvement of PNKP in the alternative OFM pathway, a FEN1 inhibitor (FEN1i) was employed to obstruct the primary OFM pathway. This approach allowed us to observe the consequences of PNKP deficiency on the secondary pathway, thereby enhancing the chances of detecting 5ʹ-OH ends. Our results indicate that while there is no notable distinction between PNKP WT and PNKP KO in DMSO-treated samples, a statistically significant increase in OF-like fragments with 5′-OH termini is observed in PNKP KO cells compared to PNKP WT upon FEN1i treatment (Fig. 3, *D* and *E*). Furthermore, consistent with this observation, CDK7 inhibition resulted in a statistically significant reduction in ^32^P incorporation regardless of PNKP status, indicating a substantial generation of 5′-OH DNA fragments during DNA replication (Fig. 3, *D* and *E*). To validate the effectiveness of CDK7i as a control, a supplementary experiment was conducted, wherein cells were labeled with BrdU for a 10-min interval prior to harvest, with some cells subjected to CDK7i treatment and others left untreated. The ensuing analysis, as depicted in Figure 3*F*, exhibited a notable decrease in BrdU incorporation in cells treated with CDK7i compared to DMSO, thereby providing additional support for the proposition that CDK7i significantly curtails DNA replication intensity. Collectively, these findings suggest the possibility of PNKP involvement in the OFM process by ensuring 5′-P DNA termini on OFs during DNA replication.

### PNKP acts in alternative OFM pathway and interacts with LIG3

**Figure 4:**
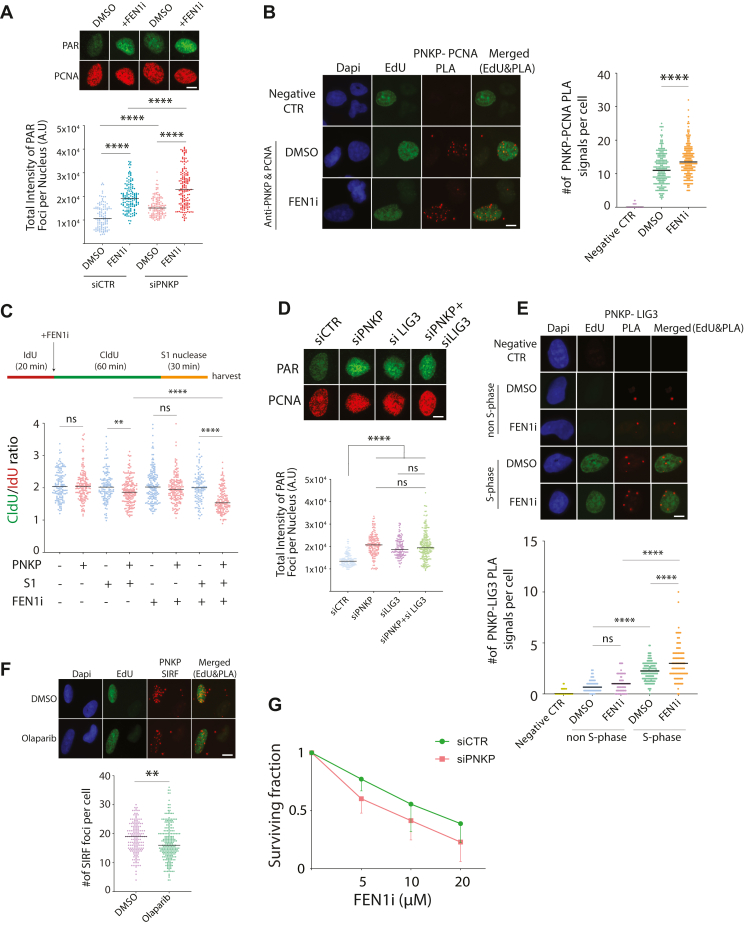
**PNKP interacts with LIG3 and plays a role in the alternative OFM pathway.***A*, Immunofluorescence experiment showing increased S-phase specific PAR chain formation (indicative of defective OFM) in response to FEN1 inhibitor (FEN1i) in U2OS cells that were treated with either control siRNA (siCTR) or siRNA against PNKP (siPNKP) 48 h before the experiment. (*Top*) shows the representative images. (*Bottom*) shows the quantifications of PCNA-positive cells. Cells were treated with a 10 μM PARG inhibitor (PARGi) for 20 min before fixation to block PAR chain removal. At least 100 cells were analyzed for each condition. Similar results were obtained from two independent experiments. The median values are marked on the graph. *B*, PLA assay with U2OS cells shows the interaction between PCNA and endogenous PNKP. Indicated cells were treated with a FEN1i, and all cells were pulse-labeled with EdU to identify those in the S-phase. Only EdU-positive cells representing cells in the S-phase were quantified. PNKP alone antibody was used for the negative control. (*Left*) shows representative images. (*Right*) shows the quantifications. Two independent experiments were performed, and more than 130 cells were analyzed for each condition in each experiment. The graph represents the data from two independent replicates. *C*, DNA fiber with S1 nuclease treatment was performed using HCT116 WT (PNKP^+^) and PNKP KO (PNKP^-^) cells. Indicated cells were treated with 10 μM FEN1i and 20 U/ml S1 nuclease for an indicated amount of time. The quantification of the ldU tract length is presented, with the median values marked on the graph. At least 100 fibers were measured per condition. The graph is representative of two independent experiments. A schematic representation of the assay conditions is presented at the top of graph (*D*) similar to (*A*), except cells were treated with either siRNA PNKP (siPNKP), LIG3 (siLIG3), or both PNKP and LIG3 (siPNKP + siLIG3) 48 h before the experiment. *E*, similar to (*B*), except the interaction between PNKP and LIG3 was studied in both S-phase (EdU positive) and non-S-phase (EdU negative) cells. Three independent experiments were performed, and more than 100 cells were analyzed for each condition in each experiment. The graph represents the data from three independent replicates. *F*, SIRF assay was used to assess the recruitment of PNKP on nascent DNA. All cells were pulse-labeled with EdU. Only EdU-positive (S-phase) cells were quantified. Cells were treated with DMSO or 10 μM Olaparib. (*Left*) shows the representative images. (*Right*) shows the quantifications. Two independent experiments were performed, and more than 100 cells were analyzed for each condition in each experiment. The graph represents the data from two independent replicates. *G*, a colony formation assay was performed using U2OS cells that were transfected with either control siRNA (siControl) or with siRNA against PNKP (siPNKP) 48 h before the experiment. The FEN1 inhibitor (FEN1i) was added at the specified concentrations for 2 days. For all quantifications in this figure: Mann-Whitney Unpaired test was performed in Prism to determine statistical significance. *Asterisks* depict statistically significant differences: ns (non-significant), ∗ (*p* < 0.05), ∗∗ (*p* < 0.01), ∗∗∗ (*p* < 0.001), ∗∗∗∗ (*p* < 0.0001). The scale bar is 10 μm.

The knockdown of PNKP seems to primarily affect 5′-OH termini only when a FEN1i is present (Fig. 3, *D* and *E*). Therefore, we aim to investigate whether PNKP is involved in the backup pathway for OFM, which includes PARP1, XRCC1, and LIG3 (18). To establish PNKP's role in this alternative pathway, we performed several experiments utilizing immunofluorescent microscopy and proximity ligation assays (PLA). Initially, we found that inhibiting FEN1 significantly increased S-phase-specific PARylation levels in PNKP WT cells, aligning with FEN1's role in OFM (18). Notably, this increase in PAR levels was much higher in PNKP deficient cells compared to PNKP WT cells (Fig. 4*A*), indicating that PNKP and FEN1 function in distinct OFM pathways. Next, we used the PLA assay to explore whether the interaction between PNKP and PCNA intensifies when the main OFM pathway is compromised. EdU-positive cells, which are marking S-phase cells, showed that the PNKP-PCNA interaction increased following treatment with the FEN1i compared to DMSO-treated controls (Fig. 4*B*). To assess whether single-strand DNA (ssDNA) gaps increase in PNKP-depleted cells as an indication of unligated OFs, we performed a modified DNA fiber assay which includes an S1 nuclease treatment (47). S1 nuclease selectively cleaves post-replicative ssDNA gaps created at RFs, resulting in shorter DNA fragments (47). As presented in Figure 4*C*, consistent with a previous report (48), FEN1i did not appear to induce measurable DNA gaps in PNKP-proficient cells. However, PNKP-depleted cells displayed a significant increase in ssDNA gaps after S1 nuclease, in comparison to PNKP-proficient cells (Fig. 4*C*). Notably, inhibition of FEN1 further exacerbates this observed effect (Fig. 4*C*). These results collectively indicate an enhanced interaction between PNKP and PCNA, along with exacerbated defects in OFM in PNKP-deficient cells when the primary OFM pathway is disrupted, implying that PNKP operates through an alternative OFM pathway.

We then sought to determine whether PNKP and LIG3 are part of the same alternative OFM pathway. Our experimental findings indicate that the reduction of either PNKP or LIG3 results in a marked elevation in S-phase-specific PARylation levels. Importantly, simultaneous depletion of both PNKP and LIG3 did not lead to a further increase in PARylation levels (Fig. 4*D*), suggesting that the two proteins work together within the same OFM pathway. Previous studies have shown that PNKP is associated with DNA ligase III in the context of SSBR and alternative NHEJ (49, 50). Given their likely cooperative role, we also examined whether PNKP and LIG3 associate during S-phase. Using PLA analysis coupled with EdU click chemistry (to label S-phase cells) with specific antibodies for both proteins, we compared PLA signals between S-phase and non-S-phase cells. Consistent with biochemical data, we confirmed that PNKP and LIG3 interact in unperturbed cells, with this interaction being enhanced in S-phase cells (Fig. 4*E*). Furthermore, FEN1 inhibition increased this interaction in S-phase cells, supporting the conclusion that PNKP and LIG3 work together in the alternative OFM pathway (Fig. 4*E*).

A previous study using a cell-free system generated from *Xenopus* egg extracts, which were depleted of LIG1 and PARP1, demonstrated that XRCC1 and LIG3 were not recruited to chromatin (18, 51). This lack of recruitment resulted in ineffective ligation of OFs. We aimed to determine if PNKP recruitment to RFs depends on PARP1 by utilizing the *in situ* protein interaction with nascent DNA replication forks (SIRF) method (52). SIRF is a modified PLA assay that incorporates 5′-ethylene-2′-deoxyuridine (EdU) click chemistry to label nascent DNA at RFs, allowing for sensitive, quantitative analysis of protein interactions at an individual cell level. Our findings demonstrate that PNKP is recruited to active RFs and that this recruitment decreases with treatment with the PARP1 inhibitor Olaparib (Fig. 4*F*). Finally, we evaluated the sensitivity of PNKP-depleted cells to FEN1 inhibition *via* a colony formation assay. As illustrated in Figure 4*G*, PNKP-depleted cells displayed slightly heightened sensitivity to FEN1 inhibition. In conclusion, our results suggest that PNKP collaborates with LIG3 to facilitate the alternative OFM process.

### Phosphorylation of PNKP by CDK1/2

**Figure 5:**
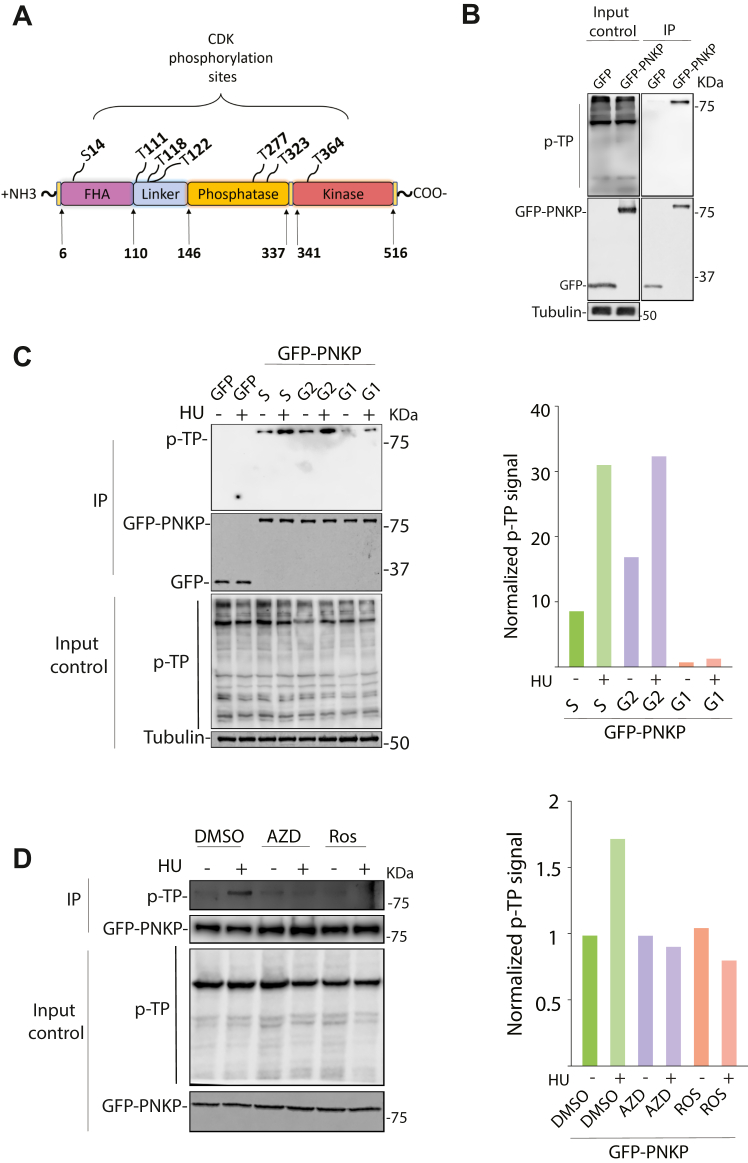
**CDK1/2 mediates PNKP phosphorylation in cells.***A*, schematic depiction of PNKP amino acid sequences with the predicted CDK phosphorylation sites labelled. *B*, IP experiments using the GFP selector beads in HEK293T cells expressing either GFP or GFP-PNKP showed that PNKP is constitutively phosphorylated *in vivo*. IP extracts were immunoblotted using an anti-phosphorylated threonine antibody followed by a proline (p-TP) as indicated. *C*, effect of cell cycle on PNKP phosphorylation. HeLa cells expressing GFP or GFP-PNKP were synchronized by a double-thymidine block and released for different time points. Cells were either left untreated or treated with HU (2 mM, 2 h). (*Left*) Cell extracts were prepared as described in Materials and Methods and immunoblotted as indicated. (*Right*) quantification of the p-TP immunoreactive bands was carried out by Image Studio software and normalized to each sample’s GFP expression and plotted as indicated. *D*, IP experiments using the GFP selector beads on extracts isolated from cells expressing either GFP or GFP-PNKP that were either left untreated or treated with HU (2 mM, 2 h). 1 h before replication stress induction, cells were treated with DMSO, AZD5438 (AZD, 10 μM), or Roscovitine (ROS, 20 μM). *Left*, IP lysates were immunoblotted as indicated. *Right*, quantifications of the p-TP signal was obtained using Image Studio software and normalized to the GFP signal in each sample.

Our results so far indicate that PNKP is recruited during DNA replication and is required for DNA RF dynamics and OFM. However, it remains unclear how the function of PNKP is controlled during DNA replication. Given that DNA replication and OFM are specific to S phase, we examined the possibility that cyclin-dependent kinases (CDKs) prevent aberrant fork progression by directly regulating PNKP. The consensus sequence of a CDK substrate is [S/T∗] PX[R/K] (6). *In silico* analysis revealed seven potential CDK consensus phosphorylation sites in human PNKP (S14, T111, T118, T122, T277, T323, and T364) (Fig. 5*A*). We utilized an antibody recognizing phosphorylated CDK substrates to confirm that PNKP is a CDK substrate. HEK293 cells were transfected either with a GFP vector or a vector expressing GFP-tagged PNKP followed by GFP immunoprecipitation (IP) under stringent conditions. Western blot analysis using an anti-phosphorylated threonine followed by a proline (p-TP) antibody CDK substrate antibody showed an immunoreactive band at the same position as GFP-PNKP (Fig. 5*B*). No signal was observed in IP eluates prepared from cells transfected with a GFP vector. These data revealed that PNKP is phosphorylated at one or more of the potential p-TP sites.

Next, we wished to determine whether PNKP is phosphorylated in a cell cycle- and replication stress-dependent manner. To this aim, cells were synchronized by a double-thymidine block at the G1/S transition and released for various time points to traverse the cell cycle. PNKP phosphorylation was examined in mock-treated or in cells treated with hydroxyurea (HU), which depletes nucleotide pools and stalls RFs (53). We found that PNKP is mainly phosphorylated during the S and G2 phases of the cell cycle (Fig. 5*C*). It is noteworthy that upon treatment of cells with HU, an increase in PNKP phosphorylation was observed across all phases of the cell cycle, with the highest induction observed in populations of cells that are enriched in the S and G2 phases (Fig. 5*C*). DNA content analysis confirmed that the cells were enriched for the indicated cell cycle stages by the synchronization protocol (Fig. S2*A*). The CDK family consists of many members participating in cell cycle regulation (54). Pharmacological inhibition of CDK1/2 using 10 μM AZD5438 (AZD) (55) or 20 μM Roscovitine (ROS) (56) reduced PNKP phosphorylation (Fig. 5*D*). These inhibitors inhibit the activity of CDKs 1 and 2, the primary CDKs responsible for advancing cells through the S and G2 phases (54). These results indicate that CDK1/2 phosphorylates PNKP, and this modification is stimulated upon exposure to replication stress.

### Phosphorylation of PNKP at five different sites in the linker and phosphatase domains

**Figure 6:**
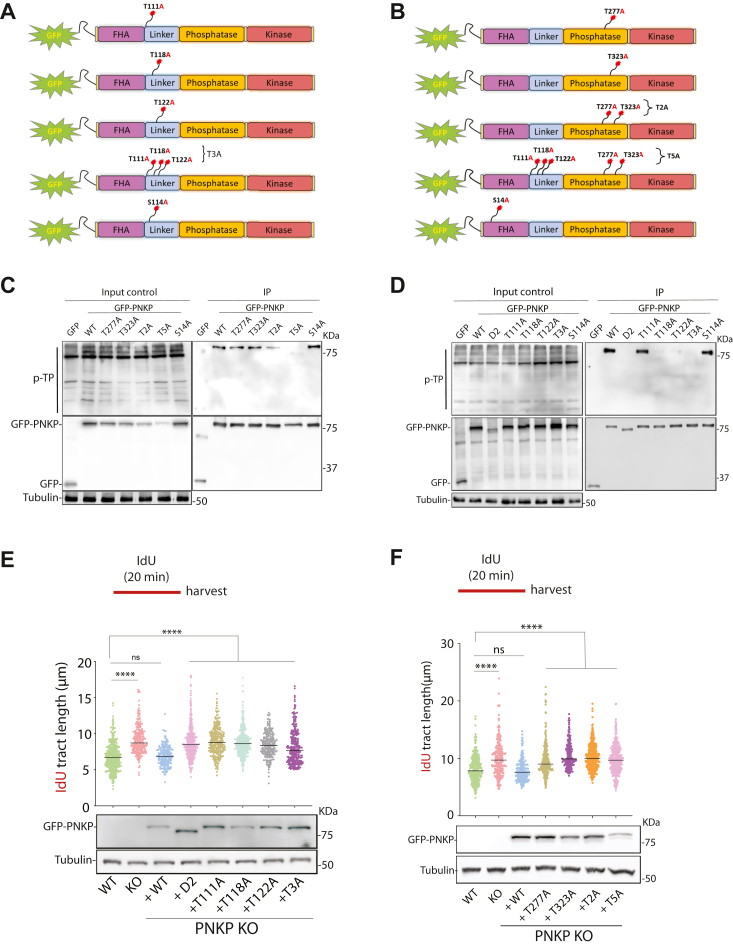
**PNKP is phosphorylated by CDK on multiple sites within linker and phosphatase domains.***A* and *B*, schematic depictions of the GFP-tagged PNKP phosphorylation mutants used in *C* and *D*. *C* and *D*, IP experiments comparing the p-TP level in cells expressing different mutants of PNKP depicted in *A* and *B*. GFP selector IP lysates were immunoblotted as indicated. *E* and *F*, DNA fiber analysis was performed in PNKP KO2 cells expressing different PNKP mutants. Cells were labeled with IdU for 20 min and harvested. The quantification of the IdU tract length is presented, with the median values marked on the graph. Between 120 to 300 fibers were measured per condition. The graphs are representative of three independent experiments. *Asterisks* indicate statistical significance (Mann–Whitney test, two-sided): ns (non-significant), ∗∗∗∗ (*p* < 0.0001). A schematic representation of the assay conditions is presented at the top of each graph. Immunoblots showing the expression levels of each PNKP expression construct are shown below. Tubulin served as a loading control.

Having established that PNKP is phosphorylated by CDK1/2, we next sought to determine which particular S/T residue(s) of PNKP are phosphorylated (Fig. 5*A*). To elucidate which domain(s) of PNKP includes potential phosphorylation sites, we used an internal deletion panel of GFP-tagged PNKP constructs (Fig. 2*B*). Despite similar immunoprecipitation of each construct from HEK293T cells, mutants D1, D2, and D3, unlike D4, showed a pronounced reduction of phosphorylation (Fig. S2*B*). Next, using site-directed mutagenesis, we mutated each phosphorylation sites in the FHA motif (S14A), linker domain (T111A, T118A, T122A, S114A) and T3A (T111A, T118A, T122A), phosphatase domain (T277A, T323A), and T2A (T277A, T323A), and T5A (T111A, T118A, T122A, T277A, T323A) which targets all phosphorylation sites in the linker and phosphatase domains (Fig. 6, *A* and *B*). To identify which of these putative sites might be necessary for PNKP phosphorylation, HEK293T cells were complemented with the PNKP constructs mentioned above, immunoprecipitated, and probed with a p-TP antibody. Wild-type PNKP (PNKP WT) served as a positive control. Compared to PNKP WT, a slight reduction in phosphorylation was observed with the T111A and T2A mutants (Fig. 6, *C* and *D*). We observed that individual threonine (T) to alanine (A) mutations of T227, and T323 had minimal impact on PNKP phosphorylation, while mutations of T118 and T122, as well as T3A and T5A, eliminated PNKP phosphorylation detection by the p-TP antibody (Fig. 6, *C* and *D*). Our data established that PNKP undergoes phosphorylation at multiple sites in cells, with the linker domain being the primary location and the phosphatase domain being the secondary location. Notably, Clustal Omega analysis of PNKP orthologues revealed that these CDK phosphorylation sites, except T118 and T277 (conserved across different species except mouse), are conserved among mammalian orthologues of PNKP (Fig. S3). Thus, our results suggest that CDKs play a major role in mediating PNKP phosphorylation at T118 and T122, whereas T111, T277, and T323, serve as secondary phosphorylation sites.

### Phosphorylation of PNKP is essential for the function of PNKP at RFs

**Figure 7:**
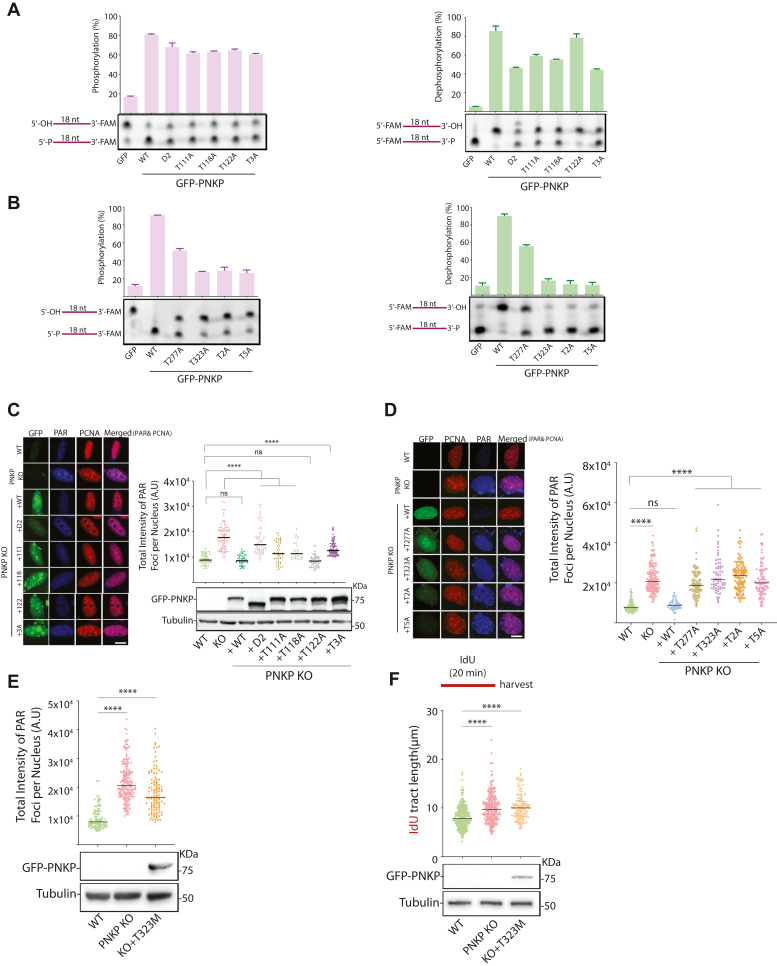
**CDK-mediated phosphorylation of PNKP is essential for its function in OFM.***A* and *B*, PNKP kinase and phosphatase assays. PNKP mutants were expressed and isolated from HEK293 cells. (*Pink bars*, located on the *left*) PNKP kinase assays were performed to assess the kinase activity of phosphorylation mutants of PNKP. (*Green bars*, located on the *right*) PNKP phosphatase assays were conducted to study the effect of each phosphorylation mutant on PNKP’s phosphatase activity. *C* and *D*, PNKP phosphorylation is required for its function in OFM. Immunofluorescence experiment showing increased PAR chain formation in PNKP KO cells reconstituted with different PNKP phosphorylation mutants. Cells were treated with a PARG inhibitor (PARGi, 10 μM) for 20 min before harvesting to block PAR chain removal. Quantification of the PAR signal in PCNA foci-positive cells are shown as well as the expression levels of each PNKP phosphorylation mutant. Tubulin serves as a loading control. Between 50 to 150 cells were analyzed for each condition. Similar results were obtained from n = 2 independent experiments. The median values are marked on the graph. To determine statistical significance, unpaired Student’s t-tests were performed in Prism. *Asterisks* depict statistically significant differences: ns (non-significant), ∗∗∗∗ (*p* < 0.0001). *E*, immunofluorescence experiment showing increased PAR chain formation in PNKP KO cells reconstituted with T323M PNKP phosphorylation mutant. Cells were treated with a PARG inhibitor (PARGi, 10 μM) for 20 min before fixation to block PAR chain removal. Quantifications of the PAR signal in PCNA-positive cells are shown. Similar results were obtained from n = 2 independent experiments. The median values are marked on the graph. The immunoblot experiment showing the expression levels of each PNKP expression construct is shown below. Tubulin served as a loading control. To determine statistical significance, unpaired Student’s t-tests were performed in Prism. *Asterisks* depict statistically significant differences: ∗∗∗∗ (*p* < 0.0001). *F*, DNA fibre analysis was performed in PNKP KO cells expressing PNKP T323M phosphorylation mutant. Cells were labelled with IdU for 20 min and harvested. The quantification of the IdU tract length is presented, with the median values marked on the graph. Between 100 to 300 fibres were measured per condition. The graph is representative of pulled data from three independent experiments. *Asterisks* indicate statistical significance (Mann–Whitney test, two-sided): ∗∗∗∗ (*p* < 0.0001). A schematic representation of the assay conditions is presented at the top of the graph. Immunoblot showing the expression levels of each PNKP expression construct is shown below. Tubulin serves as a loading control. The scale bar is 10 μm.

Having established that PNKP is phosphorylated at multiple residues within the linker and phosphatase domains, we next investigated the impact of PNKP phosphorylation on RF dynamics. All individual CDK site mutants as well as T2A, T3A, and T5A showed longer IdU tracts than control cells (Fig. 6, *E* and *F*). Interestingly, we observed that the expression of the T5A mutant is lower, compared to WT and other point mutants (immunoblots of Fig. 5, *E* and *F*). Except for the cellular expressed T122A mutant, which exhibited normal kinase and phosphatase activities, all PNKP phosphorylation mutants displayed reduced phosphatase/kinase activity levels ranging from approximately 25% to 80% reduction (Fig. 7, *A* and *B*). Therefore, one potential mechanism to explain the longer IdU tract length in PNKP KO1 cells reconstituted with these mutants is that they are defective in phosphatase/kinase activities. We then investigated the impact of PNKP phosphorylation on its function in OFM by measuring PAR chain formation in PNKP KO cells expressing PNKP-WT, -T111A, -T118A, -T122A, -T277A, -T323A, T2A, -T3A, or T5A upon acute inhibition of PARG. The D2 mutant served as a control. Apart from T122A, none of the PNKP phosphorylation mutants were able to rescue the enhanced PAR chain formation observed in PNKP KO cells (Fig. 7, *C* and *D*). The enhancement in PAR signal upon the expression of these mutants was clearly due to the loss of PNKP phosphorylation, as this defect was rescued in PNKP KO1 cells expressing PNKP WT. Together, these results indicate that PNKP phosphorylation mediates its function in DNA replication and OFM.

### T323 is a clinically important CDK phosphorylation site in PNKP

Mutations in PNKP have been associated with three neurological diseases: AOA4 (25), a variant of the hereditary peripheral neuropathy CMT2B2 (26, 57) and the autosomal recessive neurodevelopmental disorder MCSZ (24, 58, 59, 60). Recently, a young MCSZ patient was reported with a cerebellar high-grade brain tumor, glioblastoma multiforme (GBM) (60). This patient had two novel point mutations in PNKP. One in the FHA domain (P101L) and the other in the phosphatase domain (T323M). Whilst P101L does not significantly affect the enzymatic activity of PNKP, T323M dramatically reduces the phosphatase and kinase activity of PNKP (25, 60, 61). Since T323 is a CDK phosphorylation site, we wondered if its mutation to methionine, similar to alanine, affects the role of PNKP in DNA replication. First, we tested the level of PARylation in PNKP KO cells transfected with GFP-PNKP-T323M as a readout for OFM defects. The results demonstrated a high level of PARylation in PNKP KO cells with T323M mutation (Fig. 6*E*). Furthermore, the DNA fibre data showed that PNKP KO cells complemented with GFP-PNKP-T323M mutant are not able to rescue the increased replication speed (Fig. 7*F*). These results indicate that T323M mutation, similar to T323A, impairs the function of PNKP in DNA replication and OFM.

## Discussion

Many factors function to stabilize and protect stalled RFs and prevent their collapse into DSBs (62, 63). PNKP is a DNA repair enzyme that restores 5′-phosphate and 3′-hydroxyl groups to DNA ends, making them amenable for subsequent end processing and ligation (21, 64). The enrichment of PNKP at RFs identified in proteomic screens (28, 29) and its DNA end-processing activities led us to hypothesize that it is involved in DNA replication. Several lines of our data support this hypothesis. First, we found that PNKP is enriched at the RFs and colocalizes and interacts with the replication protein PCNA and nascent DNA. Second, the kinase and phosphatase structural domains are required for PNKP to restrain RF progression *in vivo*. Third, we show that CDK1/2 phosphorylates PNKP in the S and G2 phases of the cell cycle and this phosphorylation is stimulated in response to HU-induced replication stress. Finally, our data indicated increased levels of 5′-OH DNA termini in PNKP KO cells. We propose a model whereby PNKP is essential for RF progression under unperturbed conditions. Prior investigations have hinted at the formation of OF-like DNA structures featuring 5′-OH termini during DNA replication (39, 40). Our findings substantiate the presence of these OF-like DNA structures with 5′-OH termini and elucidate the indispensable role of PNKP in their restoration. The deficiency of PNKP results in aberrations in replication dynamics, manifesting as accelerated replication progression and S-phase-specific hyper-PARylation events.

PNKP plays essential roles in SSBR, BER, and NHEJ (21, 65, 66), and these functions are often regulated by posttranslational modifications. For example, PNKP is phosphorylated at serine 114 (S114) by ataxia-telangiectasia mutated (ATM) in response to IR and by the DNA-dependent protein kinase (DNA-PK) and ATM on serine 126 (S126) (67, 68). Interestingly, ATM-dependent phosphorylation of PNKP at S114 and 126 in response to oxidative DNA damage increases PNKP stability by inhibiting ubiquitylation-dependent proteasomal degradation of PNKP (67). In addition to the canonical function of PNKP in DNA repair, we found here that PNKP-deficient cells showed defects in OFM, consistent with a new role for PNKP in DNA replication. However, how PNKP function is regulated in the context of DNA replication is not known. We found that CDK1/2 mediates the phosphorylation of PNKP at multiple sites, including T111, T118, T122, T277, and T323 residues during the S and G2 phases of the cell cycle. Phosphorylation of these sites promotes replication progression and OFM. The interplay between CDK1/2 and PNKP is not surprising, as in the context of DNA end resection similar mechanisms exist in the regulation of CtIP by CDKs (69). CtIP is phosphorylated at multiple sites by CDKs, which promote DNA resection by facilitating its interactions with NBS1 and phosphorylation by ATM (69). Interestingly, while some of the primary CDK phosphorylation sites may not directly impact the intrinsic enzymatic activities of PNKP, they could play a crucial regulatory role in modulating its interactions with other cellular factors. These interactions may be indispensable for PNKP to fully exert its function in the context of RF dynamics. As the regulation of DNA repair processes often involves complex and interdependent networks of proteins, it is plausible that these mutants may fail to engage in the necessary protein-protein interactions required for efficient DNA repair processes despite retaining some enzymatic activity.

Mutations in PNKP can cause several neurological diseases, including MCSZ and AOA4 (25). Furthermore, a recent report identified an MCSZ patient with GBM that was reported to have two somatic PNKP point mutations, potentially linking MCSZ with cancer development (60). The two PNKP mutations found in the patient are C302T, which causes a proline-to-leucine change at position 101 (P101L) in the FHA domain, and C968T, which causes a threonine-to-methionine change at position 323 (T323M) in the phosphatase domain of PNKP protein. The latter mutation has recently also been identified in a Dutch MCSZ patient (70). Interestingly, T323 is among the five sites identified in PNKP to be phosphorylated in a CDK-dependent manner. We recently showed that the PNKP T323M mutant had weaker DNA kinase and phosphatase activities (60). Consistent with this notion, we found that PNKP KO cells complemented with GFP-PNKP T323M could not rescue the OFM deficiency observed in PNKP KO cells (Fig. 6, *E–G*). One intriguing scenario for the observed results could be that CDK-mediated phosphorylation of PNKP on the T323 residue regulates the enzymatic activities of PNKP. However, there is still a necessity to further characterize the regulation of PNKP in cells. While the entire genome of the patient wasn't sequenced, making it difficult to pinpoint the timing of mutations, it's plausible that the patient's mutant PNKP-induced impairment in DNA replication occurred early on and, in combination with known pediatric glioma-associated mutations like ATRX and TP53 (71), contributed to the initiation and progression of the brain tumor. Further research is needed to explore this potential link.

LIG3 forms a complex with its counterpart, XRCC1 and PNKP, playing crucial roles in SSBR and BER (50, 72, 73). Additionally, there is evidence suggesting that LIG3 could undertake the ligation of OFs in the absence of LIG1 (18, 74). Notably, XRCC1 has been observed to interact and colocalize with PCNA during the S phase (74). The approximately 20% increase in RF speed seen in PNKP KO cells resembles the effect observed in cells lacking DNA replication factors crucial for OFM, such as FEN1 and LIG1 (36, 37). In line with this concept, a recent study has implicated the PARP1-XRCC1-LIG3 pathway as a novel mechanism for alternative OFM, operating in parallel with the FEN1-LIG1 pathway (18, 36). Depletion of PARP1 also led to accelerated RF progression under normal conditions (36). Multiple lines of evidence substantiate that PNKP operates in the alternative OFM pathway alongside XRCC1, PARP1, and LIG3: Firstly, we demonstrated that PNKP interacts with PCNA in S-phase cells and this interaction is heightened when FEN1 is inhibited. Secondly, PNKP colocalizes with LIG3 in S-phase cells, hinting at potential cooperation between PNKP and LIG3. Secondly, PNKP-deficient cells display significantly higher PARylation levels, and knocking down LIG3 in a PNKP-depleted background did not further elevate the PARylation level. Thirdly, PNKP-deficient cells exhibit the generation of ssDNA gaps in nascent DNA, which is further exacerbated by FEN1 inhibition. Lastly, similar to XRCC1 and LIG3, PARP1 inhibition compromises the recruitment of PNKP into active RFs. Collectively, these findings suggest that PNKP operates within the same pathway alongside PARP1-XRCC1-LIG3 in the alternative OFM.

In conclusion, our study has unveiled novel functions of PNKP in the context of DNA replication and OFM. Further research is needed to elucidate the mechanisms underlying the generation of DNA possessing 5′-OH termini during OFM. One plausible hypothesis to account for this phenomenon is that DNA strand breaks with 5′-OH termini serve as transient structures during OFM, being purposely produced to hinder the joining of OFs for a short period. This pause would allow mismatch repair processes to differentiate between parental and newly synthesized DNA strands. In PNKP-proficient cells, PNKP together with XRCC1/LIG3 could expedite DNA ligation following PNKP action, thus facilitating the joining of OFs. In the absence of PNKP, cells have defects in OFM and accumulate unligatable DNA with 5′-OH termini. Importantly, Tsukada *et al.* (https://doi.org/10.1101/2021.07.29.452278) recently observed the unexpected function of PNKP during OFM, mirroring our results. Giacomini *et al.* showed an augmented association between PNKP and mutant H3.3 at damaged RFs (75). This newly discovered role of PNKP is an autonomous oncogenic mechanism that contributes to genome instability and stimulates tumor cell growth in pediatric high-grade gliomas (pHGG). These collective findings provide compelling evidence for the critical involvement of PNKP in DNA replication (reported here) and in pHGG pathogenesis (75) and offer potential avenues for targeted therapeutic interventions against this aggressive pediatric brain tumor.

## Experimental procedures

### Plasmids and siRNA transfection

All siRNAs that were used in this study were obtained from Sigma, Dharmacon, and Qiagen (Table S1). siRNA transfections were performed according to the manufacturer’s instructions; all siRNA transfections were performed with 20 to 40 nM siRNA using RNAiMax (Invitrogen) as a transfection reagent. All point mutants were generated using the Q5 site-directed mutagenesis kit (New England Biolabs) and verified by Sanger sequencing (Table S2). The GFP-PNKP WT and deletion mutant constructs (D1-D4) were kind gifts from Dr Mikio Shimada (Tokyo Institute of Technology, Tokyo). ΔP^−^, ΔK^−^ and, DD PNKP mutants were generated in our lab previously (76). The constructs for CRISPR knockout of PNKP in HCT116 and HeLa cells were previously described (33). His-PNKP was expressed and purified in *E. coli* as previously described (22).

### Cell culture

HCT116, HeLa, and U2OS cells were purchased from ATCC and cultured in a DMEM or DMEM/F12 medium containing 10% FCS at 37 °C and 5% CO_2_. Hydroxyurea was purchased from Sigma, dissolved in water, and stored at −20 °C. The information about the inhibitors used in the study are listed in Table S3. All inhibitors were purchased from Millipore-Sigma or Selleck Chemicals and dissolved in DMSO (or, for hydroxyurea, water). Inhibitors were diluted in a warmed (37 °C) culture medium immediately before cell treatment. Vehicle controls contained only the solvent of the inhibitors diluted to the same extent. Cells were regularly tested to ensure the absence of *mycoplasma* contamination using DAPI staining.

### Immunofluorescence (IF) staining

IF staining was performed as described previously (77). For GFP-PNKP and RFP-PCNA detection, cells were transfected with GFP-PNKP and RFP-PCNA DNA constructs 24 h before the experiment. Cells were washed with cold PBS and fixed with 4% paraformaldehyde (PFA) at room temprature. Then cells were washed with PBS 3 times before mounting with Vectashield mounting medium (Vector Laboratories). For EdU (Life Technologies) immunodetection, cells were pre-extracted with cold extraction buffer (25 mM HEPES pH 7.9, 300 mM sucrose, 50 mM NaCl, 1 mM EDTA, 3 mM MgCl_2_, and 0.5% Triton X-100) (Table S4) for 3 minutes at room temperature before being fixed at the indicated incubation time points. Cells were washed with PBS once before fixation with 4% PFA (w/v) in PBS for 20 min at room temperature. Cells were washed with PBS and permeabilized in 0.5% Triton X-100 in PBS for 3 min. Cells were incubated with a primary antibody for 1 h. (Table S5). The unbound primary antibody was removed by rinsing with 0.1% Triton X-100 in PBS at room temperature followed by three washes with PBS and incubation with the appropriate secondary antibodies (Table S6) for 1 h at room temperature. Slides were then washed three times in PBS before mounting with Vectashield mounting medium (Vector Laboratories). For PAR staining, cells were incubated with 20 min 10 μM PARGi (Cat # 7771, TOCRIS-Biotechne) before harvest. Then cells were washed with cold PBS, extracted with cold extraction buffer (25 mM HEPES pH 7.9, 300 mM sucrose, 50 mM NaCl, 1 mM EDTA, 3 mM MgCl_2_, and 0.5% Triton X-100) for 3 minutes at room temperature, washed with PBS, and fixed with cold methanol for 15 min at 4 °C. Then cells were recovered in PBS at room temperature for 10 min before incubation with primary and secondary antibodies for 1 h each, at room temperature (Tables S5 and S6). Coverslips were observed using an upright fluorescence microscope (Zeiss AxioImager.Z1) with a Plan Neofluar 1.3 N.A. ×40 oil immersion objective. For EdU-PNKP and PCNA-PNKP colocalization, Plan-Apochromat 40×/1.3 Oil DIC lens on a ZEISS 710 confocal microscope with Zen 2011 software (Carl Zeiss) was used. The brightness and contrast were scaled evenly among all samples within an experiment.

### Western blotting

The cells were lysed using 1× SDS-PAGE sample buffer containing 125 mM Tris (pH 6.8), 4% sodium dodecyl sulfate, 20% glycerol, 0.1% bromophenol blue, and 5% 2-mercaptoethanol, supplemented with 1× protease inhibitor (Complete EDTA-free tablet, Roche) and 1× phosphatase inhibitor (PhosSTOP, Roche). Subsequently, the lysates were transferred onto a nitrocellulose membrane (0.2 μm) by electrophoresis at 110 V for 90 min in transfer buffer composed of 25 mM Tris-HCl, 0.2 M glycine, and 20% methanol. Following the transfer, the membranes were blocked with either 4% fish skin gelatin (FSG) or 5% skimmed milk diluted in TBS-T buffer (TBS buffer with 0.1% Tween-20) for a minimum of 1 h at room temperature, and then incubated overnight at 4 °C or for 1 h at room temperature with primary antibodies diluted in 2% FSG in TBS-T. A range of commercially available primary antibodies targeting various proteins were utilized (Table S5). Subsequently, the membranes were washed three times with TBS-T for 10 min each and then incubated with secondary antibodies conjugated to horseradish peroxidase (HRP) or labeled with infrared dye (IRDye 680RD) (all from LI-COR Biosciences) (Table S6) diluted in 2% FSG in TBS-T. Following an additional three washes with TBS-T, HRP activity was visualized using the Odyssey Fc Imaging System (LI-COR Biosciences) after the addition of the substrate for the Amersham ECL Prime reagent (Cytiva).

### GFP selector immunoprecipitation (IP)

Cells were collected and lysed in IP lysis buffer (50 mM Tris–HCl pH 7.48, 150 mM NaCl, 1% IGEPAL CA-630, 1% sodium deoxycholate, 0.1% SDS, 1 mM EDTA), 1× Complete EDTA-free protease inhibitor (Roche) and 1× PhosSTOP (Roche) for 30 min on ice. To clarify the samples, centrifugation was performed at 14000*g* at 4 °C. As input, 10% of the supernatant was collected and denatured in 2× sample buffer (50 mM Tris-HCl pH 6.8; 2% SDS; 10% glycerol; 12.5 mM EDTA; 0.02% bromophenol blue; 5% 2-mercaptoethanol) and heated for 5 min at 95 °C. Protein extracts were incubated with 30 μl of GFP selector agarose beads (Nano Tag Biotechnologies) for 1 h at 4 °C. The beads were washed three times in wash buffer (50 mM Tris–HCl pH 7.9 1% IGEPAL CA-630, 1 mM EDTA, 1M NaCl). After washing the beads, immunoprecipitated proteins were eluted in a 2× SDS-PAGE sample and denatured at 95 °C for 5 min.

### Ni-NTA pull down assay

For each sample, 25 μl Ni-NTA agarose beads (Qiagen) were equilibrated with 1 ml NETN buffer (20 mM Tris, pH 8, 1 mM EDTA, 0.5% IGEPAL CA-630 (Sigma), 500 mM NaCl) followed by centrifugation at 5000*g* for 5 min at 4 °C. The supernatant was discarded, and the beads were resuspended in 100 μl NETN buffer. Afterward, 1 μg His-PNKP and 1 μg purified PCNA (cat #H00005111-P01, Abnova) were added to 100 μl beads- NETN suspension and incubated at 4 °C, 1200 rpm for 1 h. 1 μg bovine serum albumin (BSA) was used as a negative control. Then samples were centrifuged, and the pellet was washed 3 times. Finally, the pulled-down proteins were eluted in 2× SDS-PAGE sample buffer (125 mM Tris pH 6.8, 4% sodium dodecyl sulfate, 20% glycerol, 0.1% bromophenol blue, 5% 2-mercaptoethanol) and heated at 95 °C for 5 min. To confirm the purity of the proteins, 1 μg of each was loaded on a 10% polyacrylamide gel and the gel was stained with Coomassie Brilliant Blue (Bio-Rad, G-250, Cat # 1610406). To make the staining solution, 100 mg Coomassie Brilliant Blue G-250 was dissolved in 50 ml of 95% ethanol. Next, 100 ml of 85% phosphoric acid was added while stirring. After the dye was completely dissolved, double distilled water was added to 1L final volume. After staining the gel, the excess dye was removed by de-staining buffer (50% methanol, 40% acetic acid, 10% H_2_O) prior to photographing the gel.

### Isolation of proteins at nascent DNA (iPOND)

iPOND was conducted following the initial protocol with slight adjustments (78). HEK293T cells were briefly exposed to a 10 μM EdU (Life Technologies) pulse for 10 min. For thymidine chase experiments, cells underwent triple washing with a complete medium and were then incubated in a medium supplemented with 10 μM thymidine (Sigma Aldrich) for 60 min. Protein-DNA crosslinking was carried out in 1% formaldehyde for 15 min at room temperature, followed by quenching with 0.125 M glycine for 5 min and triple washing with PBS. Subsequently, cells were permeabilized with 0.25% Triton X-100 in PBS for 30 min, washed once with 0.5% BSA in PBS and once with PBS alone, and then subjected to click reaction buffer (comprising 10 mM sodium ascorbate, 20 μM biotin azide, and 2 mM Cu_2_SO4) incubation for 2 h at room temperature. For the “no-click” control sample, DMSO was utilized instead of biotin azide in the click reaction buffer. Cells were washed once with 0.5% BSA in PBS and with PBS alone before being lysed in buffer (consisting of 50 mM Tris-HCl, pH 8.0, and 1% SDS) supplemented with protease/phosphatase inhibitor (Roche). Chromatin solubilization was achieved by subjecting samples to sonication for 2 min (30 s on and 30 s off) at 4 °C using amplitude 30 on a Model 705 Sonic Dismembrator equipped with a microtip probe (Fisher Scientific). Samples were then centrifuged for 10 min at 5000*g*, and supernatants were diluted 1:1 with PBS containing protease/phosphatase inhibitors and incubated overnight at 4 °C with streptavidin-agarose beads (EMD Millipore). Beads were washed twice with lysis buffer, once with 1 M NaCl, twice with lysis buffer again, and then heated in boiling beads elution buffer in 2× SDS Sample Buffer (Life Technologies) containing 200 mM DTT for 10 min at 95 °C. Proteins were separated by electrophoresis and detected *via* Western blotting.

### Cell cycle synchronization

HEK293T cells were synchronized at the G1/S transition using a double-thymidine block method. Initially, the cells were seeded to attain a confluency of 40 to 50%, following which 3 to 4 mM deoxythymidine was introduced into the culture media for a period of 14 to 18 h (first block). Subsequently, the cells were allowed to progress through the cell cycle by undergoing two washes with PBS and then being cultured in warmed DMEM media for 12 h. A subsequent block was applied by adding 4 mM deoxythymidine to the culture media for an additional 18 h (second block). The cells were then released from synchronization by undergoing two washes with PBS and being replaced with warmed culture media for a duration appropriate for the desired cell cycle phase (*e.g.*, 3 h for S phase, 7 h for G2, and 12 h for G1 phase).

### Cell cycle analysis

Asynchronous and synchronized cells were trypsinized, washed once with PBS, and then resuspended in 100 μl of PBS. Subsequently, ice-cold 70% ethanol in 1 × PBS was added dropwise, and the samples were incubated overnight at −20 °C. Following this, the samples were washed once with PBS and treated with 100 μg/ml RNase A in PBS containing 3.8 mM sodium citrate for 30 min at 37 °C with agitation. Propidium iodide was then added to a final concentration of 50 μg/ml, and the cells were further incubated for 30 min. Flow cytometry analysis was performed on the processed samples using a BD FACSCanto II (BD Biosciences), with propidium iodide fluorescence detection implemented after gating for forward and side scatter.

### DNA fiber assays

Fibre assays were conducted following a previously described protocol with some adjustments (77). Initially, asynchronous cells were labeled with thymidine analogs (Sigma): 20 μM iododeoxyuridine (IdU) followed by 200 μM chlorodeoxyuridine (CldU). Then cells were harvested by trypsinization, washed, and suspended in 60 μl of PBS. Subsequently, 2 μl of the cell suspension was dropped onto a negatively charged slide (Denville Ultraclear), and *in situ* cell lysis was performed by adding 10 μl of lysis buffer (200 mM Tris-HCl, pH 7.5; 50 mM EDTA; 0.5% SDS). Stretching of high-molecular-weight DNA was achieved by tilting the slides at 15 to 20 °C to let gravity spread the fibers down the slides. The resulting DNA spreads were air-dried for 40 min, followed by fixation for 5 min in 3:1 methanol: acetic acid solution and refrigerated overnight (slides can be stored in the cold room for a couple of days). For immunostaining, stretched DNA fibers were denatured with 2.5 N HCl for 30 min, washed three times for 5 min each in PBS, and then blocked with 5% BSA in PBS for 60 min at room temperature. Rat anti-CldU/BrdU, chicken anti-rat Alexa 488, mouse anti-IdU/BrdU, and goat anti-mouse IgG1 Alexa 547 antibodies were utilized to reveal CldU- and IdU-labeled tracts, respectively (Tables S5 and S6). For the S1 nuclease DNA fiber, we followed the protocol precisely as developed by Martins *et al.* (47). In brief, the cells were labelled with IdU for 20 min, followed by CldU with or without FEN1i for 1 h. Following this, the nuclei were extracted and treated with S1 nuclease for 30 min prior to harvest. The remaining procedure is similar to the conventional DNA fiber described above. The labeled tracts were visualized under an upright fluorescence microscope (Zeiss AxioImager.Z1) with a Plan Neofluar 1.3 N.A. ×40 oil immersion objective, and tract lengths were measured using MetaXpress six software (Molecular Devices, LLC). A total of 100 to 300 fibers were analyzed for each condition in each experiment. Statistical analysis of the tract length was performed using GraphPad Prism.

### PLA for exogenous PNKP and PCNA

For PLA assay, U2OS cells were grown on coverslips and transfected with either GFP or GFP-PNKP plasmids 24 h prior to the experiment. On the day of the experiment, cells were 40% confluent. Cells were fixed with cold MeOH in a cold room for 20 min, recovered in PBS for 10 min at room temperature, permeabilized with 0.5% TritonX-100 in PBS for 5 min, and washed 2 times with PBS. The next steps followed the manufacturer’s protocol (Duolink In Situ- Sigma- Aldrich). PCNA and GFP Primary antibodies were used with 1:200 dilution.

### PLA for endogenous proteins and SIRF

U2OS cells were grown on coverslips to 50 to 60% confluency for PLA and SIRF assay. On the day of the experiment, cells were treated or not with Olaparib (10 μM) and Fen1i (10 μM) for a total of 1 h, and for the last 10 min, they were labeled with EdU (125 μM) to visualize replicating cells in S-phase. After treatment, cells were washed with ice-cold PBS and extracted for 3 min in RT. Next, cells were fixed with 2% PFA for 15 min at RT and allowed to recover in PBS for 10 min. Then they were permeabilized with 0.5% TritonX-100 in PBS for 15 min followed by three washes with PBS. For PCNA/PNKP PLA, cells were fixed and permeabilized with cold MeOH for 20 min in −20. Subsequently, cells were incubated with a fresh Click-it reaction cocktail (2 mM of copper sulfate, 10 μM of biotin-azide containing 1:10 Alexa 488 azide, and 100 mM of sodium ascorbate in PBS) in RT for 1 h. Following the Click-iT reaction, the next steps followed the manufacturer’s protocol (Duolink In Situ- Sigma- Aldrich). PCNA, PNKP, biotin, and LIG3 Primary antibodies were used with 1:200 dilution.

### Alkaline DNA gel electrophoresis

To determine the presence of genome-embedded ribonucleotides in nuclear DNA, alkaline gel electrophoresis of RNase-H2-treated genomic DNA was performed as previously described (79).

### PNKP phosphatase assay

The PNKP phosphatase assay involved immunoprecipitation of wild-type and mutant GFP-PNKP from cell extracts, as described previously (80). These immunoprecipitated proteins were then added to a 20 μl reaction mixture comprising phosphatase buffer (50 mM Tris-HCl, pH 7.4, 10 mM MgCl_2_, 0.1 mM EDTA, 0.1 mM spermidine, and 0.1 mM dithiothreitol) and 0.01 mM 5′-FAM labeled 3′-phosphorylated 18-nt DNA substrate (5′-/56-FAM/TAGCATCGATCAGTCCTC/3Phos/-3′; Integrated DNA Technologies, San Francisco, CA). The reaction mixtures were then incubated for 20 min at 37 °C. Subsequently, 2 μl of the incubated samples were mixed with 2 μl of 2× sequencing gel loading dye (comprising 90% formamide, 0.5% EDTA, 0.1% bromophenol, and 0.1% xylene cyanol), boiled for 10 min, and subjected to electrophoresis at 1800 V on a 12% polyacrylamide sequencing gel containing 7 M urea. The gels were scanned using a Typhoon 9400 variable mode imager (GE Healthcare), and the resulting bands were quantified using Image Quant 5.2 software (GE Healthcare).

### PNKP kinase assay

Wild-type and mutant GFP-PNKP proteins were immunoprecipitated from cell extracts following the previously described method (80). Subsequently, the immunoprecipitated proteins were added to a 20 μl reaction mixture comprising kinase buffer (80 mM succinic acid, pH 5.5, 10 mM MgCl2, and 1 mM dithiothreitol), 0.01 mM of a 3′-FAM labeled 18-nt DNA substrate bearing a 5′-OH terminus (5′-TAGCATCGATCAGTCCTC/36-FAM-3′; Integrated DNA Technologies), and 0.2 mM ATP (Sigma, Oakville, ON). The reaction mixtures were then incubated for 20 min at 37 °C. After the incubation, 2 μl of the reaction samples were mixed with 2 μl of sequencing gel loading dye (composed of 90% formamide, 0.5% EDTA, 0.1% bromophenol, and 0.1% xylene cyanol), boiled for 10 min, and subjected to electrophoresis at 1800 V on a 12% polyacrylamide sequencing gel containing 7 M urea. The gels were scanned using a Typhoon 9400 variable mode imager (GE Healthcare), and the resulting bands were quantified using Image Quant 5.2 (GE Healthcare).

### Isolation and detection of OF-like DNA fragments

Cells were subjected to a 16 h treatment with 3 mM thymidine to synchronize at the G1/S transition. Subsequently, the cells were washed twice with PBS and released to progress into S phase for 80 min. To manipulate specific cellular processes, either CDK7 inhibitor (CDK7i, PHA-793887, Selleckchem) or FEN1 inhibitor (FEN1i, MedChemExpress (MCE), Cat # HY-123834) was introduced to the media. Cell pellets were washed with PBS once and the nuclei were extracted using nuclei extraction buffer (0.5% Triton X-100, 10 mM Tris-HCl, 3 mM CaCl_2_, 250 mM sucrose, 0.1 mM DTT, 1 mM phenylmethylsulfonyl fluoride (PMSF in isopropanol)) for 10 min on ice. The nuclei were spun down at 4 °C at 14,000 rpm for 15 min, isolated, and mixed with 1.5% agarose in 1:1 (v/v) ratio. Agarose plugs containing nuclei were transferred to 2 ml Eppendorf tubes and incubated with lysis buffer (1% sodium lauroyl sarcosinate, 0.2% sodium deoxycholate, 1 mg/ml proteinase K, 100 mM EDTA) for 30 min at 37 °C, washed 3 times with washing buffer (20 mM Tris-base, 50 mM EDTA) and incubated with 0.3 mg/ml RNase A (Invitrogen, PureLink, cat # 12091021) for 30 min at room temperature. Then, the plugs were washed 3 times with 1× alkaline agarose buffer (for 1 L 10× buffer, 50 ml 10 N NaCl and 20 ml 0.5 M EDTA (pH 8.0) in H_2_O were used), placed and sealed in wells of a 1.5% alkaline agarose gel and run for 2.5 h in 1× alkaline agarose buffer (81). After electrophoresis, the alkaline gel was neutralized in 1 M Tris-HCl and 1.5 M NaCl for 45 min and stained with SYBR gold (Invitrogen, cat # 11494) for at least 20 min. DNA fragments (200 nt and smaller) were extracted from the gel using QIAEX II gel extraction kit (Qiagen cat # 20051) and radiolabeled in 20 μl reaction mixture containing kinase buffer (80 mM succinic acid (pH 5.5), 10 mM MgCl_2_, and 1 mM dithiothreitol), 1.65 pmol of [γ-^32^P] ATP (3000 Ci/mmol, Revvity) and 3 μg of recombinant human PNKP (60) by incubation for 45 min at 37 °C. Subsequently, samples were loaded on an 8% urea gel (82) (for a final volume of 20 ml, 9.6 g urea, 4 ml 40% acrylamide/bisacrylamide, 2 ml 10× TBE, 200 μl 10% ammonium persulfate, 8 μl TEMED) and the gel was scanned on a Typhoon 9400 variable mode imager (GE Healthcare). The resulting smears were quantified by ImageJ 1.53t.

### Image processing and statistical analysis

All immunofluorescence (IF) images were analyzed using the cell analysis tool in Imaris software (Oxford Instruments). The region of interest (ROI) was defined for colocalization studies to identify the nuclei. Subsequently, a separate channel was created for the colocalized signal, from which foci counts were analyzed and compared. Composite figures of collected images were compiled using Adobe Photoshop 2022 (Adobe) and labeled in Illustrator 2017 (Adobe). All microscopic and immunoblot images underwent brightness and contrast adjustments in Adobe Photoshop before being arranged and labeled in Adobe Illustrator. Bar graphs and scatterplots were generated in Prism (Graphpad), displaying the mean and standard deviation (error bars). Statistical significance was determined using two-tailed, unpaired, non-parametric Student’s t-tests (Mann–Whitney), Two-way ANOVA, Tukey’s multiple comparisons, and one-way ANOVA with multiple comparisons in Prism. Asterisks indicate statistically significant differences: ns (not significant), ∗ (*p*<<0.05), ∗∗ (*p*<<0.01), ∗∗∗ (*p* < 0.001), ∗∗∗∗ (*p* < 0.0001). Schematic diagrams were created using Microsoft PowerPoint and Adobe Illustrator.

## Data availability

All data presented in this document can be found within the manuscript and accompanying supplementary files. This study produced a set of plasmids and cell lines. All materials will be made available upon reasonable request following publication.

## Supporting information

This article contains supporting information.

## Conflict of interest

The authors declare that they have no conflicts of interest with the contents of this article.
