# Supplementary material for: CDK-dependent phosphorylation regulates PNKP function in DNA replication
Source: J Biol Chem. 2024 Oct 11;300(11):107880. doi: 10.1016/j.jbc.2024.107880 (PMC11650725; doi:10.1016/j.jbc.2024.107880)
Supplement: Supplemental Tables S1–S6 [file mmc1.pdf]

SUPPLEMENTAL INFORMATION

TABLES

Supplementary Table S1: siRNAs Used

Protein Targeted	Sense Sequence (5'→3')	Manufacturer
PNKP	AGAGAUGACGGACUCCUCUUU	Dharmacon
PNKP(3'UTR)	ACAAUAAACGCUGUUUCUCCUU	Dharmacon
Ligase1	GGCAUGAUCCUGAAGCAGA[dt][dt]	Sigma
FEN1	CCAUUCGCAUGAUGGAGAA[dt][dt]	Sigma
LIG3	CCACAAAAAAAAUCGAGGATT	Dharmacon
Negative Control siRNA	N/A	Qiagen

Supplementary Table S2: Primers for Site-Directed Mutagenesis

Mutant	Primer sequences (5'→3')	Kit Used	Reference for template
T111A	F- AGAGACCCGCgCACCAGAATC<br>R- TCCCAGCGCAGGGTCAGT	Q5	N/A
T118A	F-CCAGCCAGATgCTCCGCCTGG<br>R- GATTCTGGTGTGCGGGTCTC	Q5	N/A
T122A	F- TCCGCCTGGCgCCCCTCTGGT<br>R-<br>GTATCTGGCTGGGATTCTGGTGTG<br>CG	Q5	N/A
S114A	F-<br>CACACCAGAAgCCCAGCCAGATA<br>C<br>R- CGGGTCTCTTCCCAGCGC	Q5	N/A
T277A	F- CAACGACGGCgCGCCCATATC<br>R- GCCTGCTCCTGCAGATGG	Q5	N/A
T323A	F- GCCCTTCGCCgCGCCTGAGGA<br>R-<br>AGGCCAAGGTTGAGGGCAAACAG	Q5	N/A
T323M	F-<br>CCTGCCCTTCGCCATGCCTGAGGA<br>G<br>R-<br>CTCCTCAGGCATGGCGAAGGGCA<br>GG	QuickChange II site-directed mutagenesis kit (Stratagene, La Jolla, CA)	PMID:3535484<br>5
S14A	F-<br>GTGGCTCGAGgcCCCCCCTGGGGG<br>AGC<br>R- AAGCGGCCCGGGGCCTCC	Q5	N/A

N/A: non-applicable

Supplementary Table S3: Inhibitors

**Name of the inhibitor**	**Manufacturer and Catalogue number**	**Concentration used**
AZD 5438	Selleckchem, Cat # S2621	10 µM
Roscovitine	CAYMAN CHEMICAL COMPANY	20 µM
PARG inhibitor	TOCRIS-Biotechne, Cat # 7771	10 µM
Emetine	Sigma-Aldrich, Cat # E2375	1 µM
Olaparib	Selleckchem, Cat # S1060	10 µM
FEN1 inhibitor	MedChemExpress, Cat #HY-123834	10 µM

Supplementary Table S4: Extraction Buffer for Immunofluorescence Staining

Experiment name	Buffer Composition	Conditions
Immunodetection and S1 nuclease DNA fiber	25 mM HEPES pH 7.9, 300 mM sucrose, 50 mM NaCl, 1 mM EDTA, 3 mM $MgCl_2$, 0.5% Triton X-100	ice-cold buffer 3 minutes at room temprature

Supplementary Table S5: Primary Antibodies

Antigen	Host	Manufacturer	Catalogue Number	Application
α-Tubulin	mouse	Millipore Sigma	T6074	IB (1:10000)
α-Tubulin	mouse	GeneTex	GTX628802	IB (1:10000)
PNKP (B5)	mouse	Santa Cruz Biotechnology	Sc-365724	IB(1:1000)
PNKP	rabbit	House-made	N/A	Used for IP
PNKP (PLA and SIRF)	rabbit	Invitrogen	MA5-44764	1:200
BrdU (for CldU)	rat	Abcam	ab6326	Fiber (1:200)
BrdU (for IdU)	mouse	BD Biosciences	347580	Fiber (1:200)
GFP	mouse	Santa Cruz Biotechnology	Sc-9996	IB (1:2000)
Histone H2A	rabbit	Upstate / Millipore Sigma	07-146	IB (1:5000)
Phospho-Histone H2AX at S139 (γH2AX)	rabbit	Active Motif	39118	IF (1:1000)
Phospho-Histone H2AX at S139 (γH2AX)	mouse	Millipore Sigma	05-636-I	IB (1:2000)<br>IF (1:2500)
PCNA	mouse	Santa Cruz Biotechnology	sc-56	IB (1:1000)
Ligase1	mouse	Santa Cruz Biotechnology	Sc-271678	IB (1:1000)
FEN1	mouse	Santa Cruz Biotechnology	Sc-28355	IB (1:1000)
p-TP	mouse	Cell Signaling	9391	IB (1:1000)
PAR	rabbit	Millipore	MABE1016	IF (1:1000)
LIG3	mouse	Invitrogen	MA1-23191	PLA (1:200)
Biotin	mouse	Millipore Sigma	B7653	SIRF (1.100)

IF: immunofluorescence; IB: immunoblot; Fiber: DNA fiber assay, IP: immunoprecipitation

Supplementary Table S6: Secondary Antibodies

Antibody Conjugate	Manufacturer	Catalogue Number	Application
chicken anti-rat—Alexa Fluor 488	ThermoFisher Scientific	A-21470	Fiber (1:300)
goat anti-mouse—Alexa Fluor 546	ThermoFisher Scientific	A-21123	Fiber (1:300)
goat anti-mouse—HRP	LI-COR	926-80010	IB (1:5000)
goat anti-rabbit—HRP	LI-COR	926-80011	IB (1:5000)
donkey anti-mouse—IRDye 680RD	LI-COR	926-68072	IB (1:20000)
donkey anti-rabbit—IRDye 680RD	LI-COR	926-68073	IB (1:20000)
donkey anti-mouse—IRDye 800CW	LI-COR	926-32212	IB (1:20000)
donkey anti-rabbit—IRDye 800CW	LI-COR	926-32213	IB (1:20000)
donkey anti-mouse—Alexa Fluor 488	ThermoFisher Scientific	A-21202	IF (1:1000)
donkey anti-mouse—Alexa Fluor 594	ThermoFisher Scientific	A-21203	IF (1:1000)
donkey anti-rabbit—Alexa Fluor 594	ThermoFisher Scientific	A-21207	IF (1:1000)
donkey anti-rabbit—Alexa Fluor 647	ThermoFisher Scientific	A-31573	IF (1:300)
goat anti-mouse—Alexa Fluor 647	Abcam	ab150115	IF (1:300)

HRP: horseradish peroxidase; IF: immunofluorescence; IB: immunoblot; Fiber: DNA fiber assay
