# Supplementary material for: CDK-dependent phosphorylation regulates PNKP function in DNA replication
Source: J Biol Chem. 2024 Oct 11;300(11):107880. doi: 10.1016/j.jbc.2024.107880 (PMC11650725; doi:10.1016/j.jbc.2024.107880)
Supplement: Supplemental Figures S1–S3 [file mmc2.pdf]

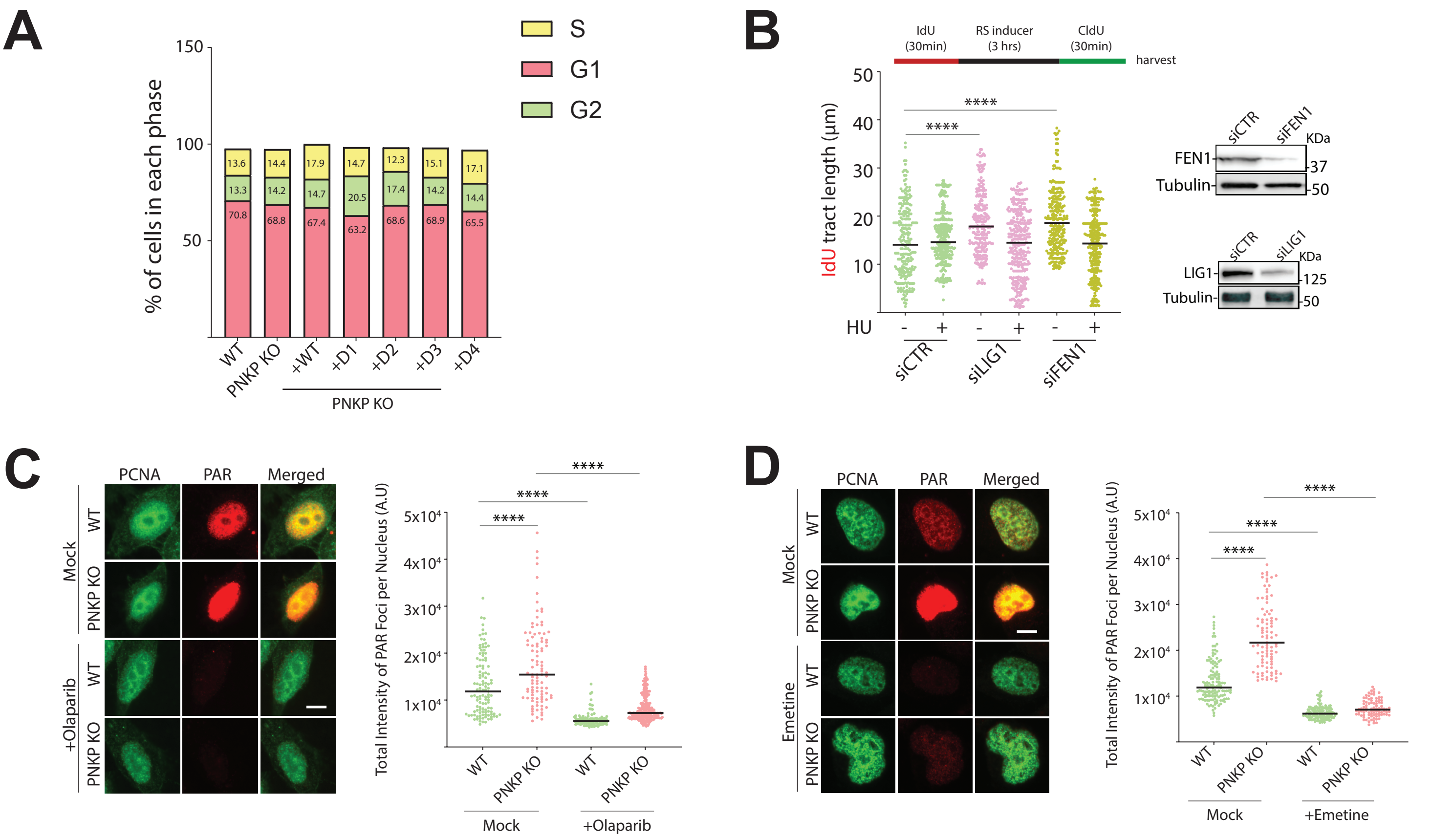

Supplemental Figure S1: Loss of PNKP does not change the cell cycle phase distribution and induces PAR formation. (A) HCT116 cells were synchronized to different phases of the cell cycle using a double-thymidine (2 mM) block. Cells were either left untreated or were treated with 2 mM HU for 2 h and processed for propidium iodide staining and DNA content analysis. Shown are the averages of 2 independent experiments. **(B)** DNA fibre analysis was performed in cells transfected with control siRNA (siCTR) or Ligase 1 siRNA (siLIG1), or FEN1 siRNA (siFEN1) for 48 h. Cells were labelled with IdU for 30 minutes, treated with 2 mM HU for 4 h, and labelled with CldU for 30 minutes. IdU track lengths were measured and plotted as indicated. Data from two independent experiments from two biological repeats, with at least 100 fibres measured per experiment. The graph is representative of at least two independent experiments. Asterisks indicate statistical significance (Mann–Whitney test, two-sided): **** ($P < 0.0001$). Lysates from cells transfected with either siCTR or siLIG1/siFEN1 were blotted for LIG1/FEN1 or Tubulin. **(C-D)** Immunofluorescence experiment showing increased PAR chain (indicative of defective OFM) formation in PNKP knockout cells (PNKP KO) or PNKP KO cells reconstituted with PNKP WT construct. Cells were treated as indicated with a PARG inhibitor (PARGi) for 20 min before harvesting to block PAR chain removal. Cells were immunostained as indicated. PCNA staining serves as a marker for the S phase. Representative images of cells were shown on the left. Quantifications of the PAR signal in PCNA positive cells were shown on the right. At least 60 cells were analysed for each condition. Similar results were obtained from anotherindependent experiments. The median values are marked on the graph. To determine statistical significance, unpaired Student's t-tests was performed in Prism. Asterisks depict statistically significant differences: **** ($P< 0.0001$). **(C)** Cells were pretreated with PARP inhibitor Olaparib for 1 hr. **(D)** Cells were pretreated with Emetine for 1 h. The scale bar is 10 μm.

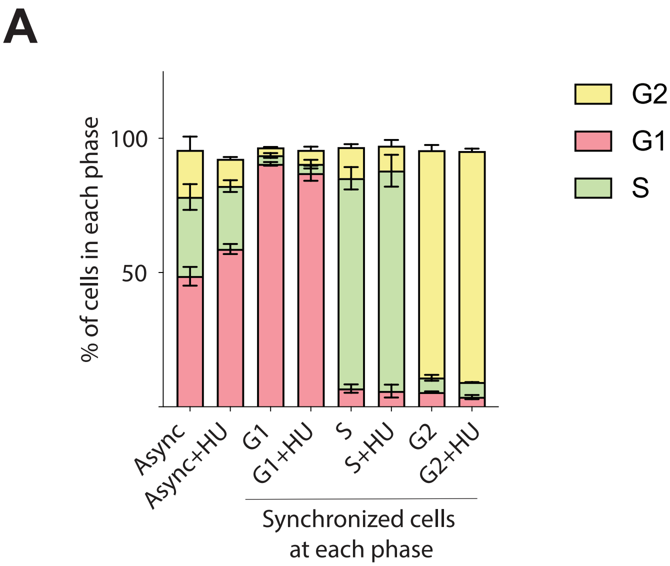

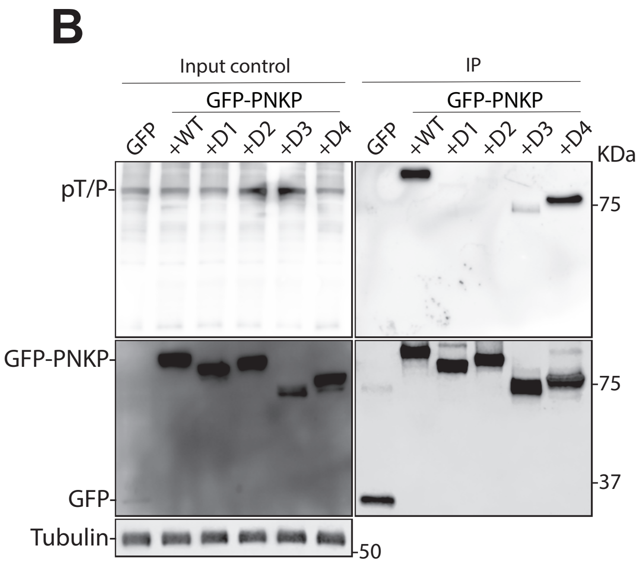

Supplemental Figure S2: **Mapping out domain requirements for PNKP phosphorylation. (A)** Cell-cycle profile of synchronized HeLa cells transfected with GFP-PNKP in the presence and absence of HU. Cells were processed for propidium iodide staining and DNA content analysis as described in the material and methods. Shown are the averages of 2 independent experiments. **(B)** Immunoprecipitation (IP) experiments using the GFP selector beads in cells expressing either GFP or GFP-PNKP truncation mutants. Lysates and elutes were prepared as described in the materials and methods and immunoblotted as indicated. PNKP phosphorylation was detected using p-TP antibody.

Mashayekhi *et al.* **Supplementary Figure 3**

A

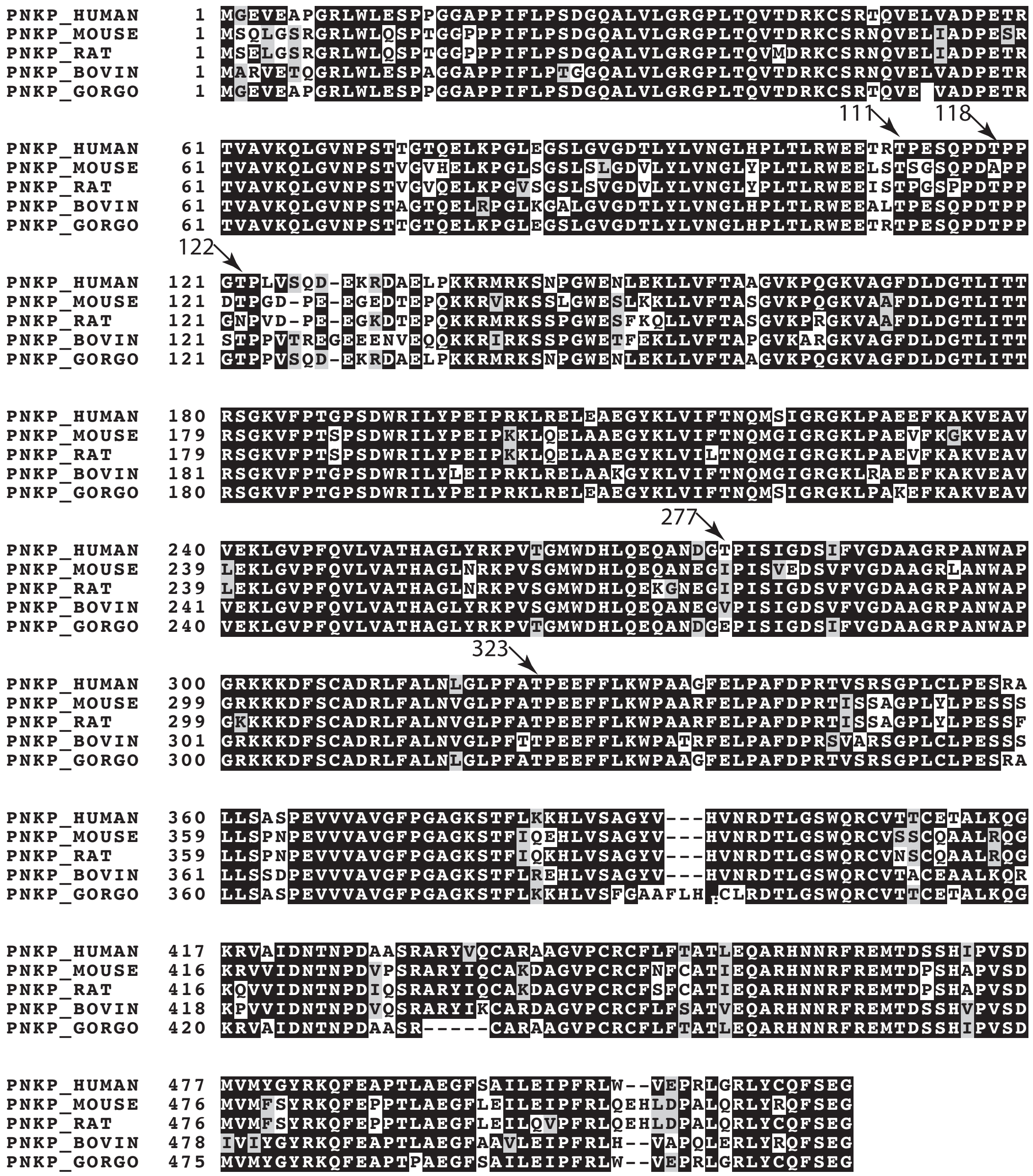

B

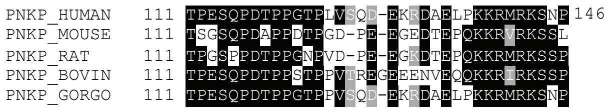

Supplemental Figure S3: **(A) Sequence alignment of the full length human PNKP with PNKP sequence from different species. (B)** The conservation of the PNKP phosphorylation sites across different species.
